# Accommodating exogenous variable and decision rule heterogeneity in discrete choice models: Application to bicyclist route choice

**DOI:** 10.1371/journal.pone.0208309

**Published:** 2018-11-30

**Authors:** Bibhas Kumar Dey, Sabreena Anowar, Naveen Eluru, Marianne Hatzopoulou

**Affiliations:** 1 Department of Civil, Environmental and Construction Engineering, University of Central Florida, Orlando, Florida, United States of America; 2 Department of Civil Engineering, University of Toronto, Toronto, Canada; Shandong University of Science and Technology, CHINA

## Abstract

The proposed research contributes to our understanding of incorporating heterogeneity in discrete choice models with respect to exogenous variables and decision rules. Specifically, the proposed latent segmentation based mixed models segment population to different classes with their own decision rules while also incorporating unobserved heterogeneity within the segment level models. In our analysis, we choose to consider both random utility and random regret theories. Further, instead of assuming the number of segments (as 2), we conduct an exhaustive exploration with multiple segments across the two decision rules. The model estimation is conducted using a stated preference data from 695 commuter cyclists compiled through a web-based survey. The probabilistic allocation of respondents to different segments indicates that female commuter cyclists are more utility oriented; however, the majority of the commuter cyclist’s choice pattern is consistent with regret minimization mechanism. Overall, cyclists’ route choice decisions are influenced by roadway attributes, cycling infrastructure availability, pollution exposure, and travel time. The analysis approach also allows us to investigate time based trade-offs across cyclists belonging to different classes. Interestingly, we observe that the trade-off values in regret and utility based segments for roadway attributes are similar in magnitude; but the values differ greatly for cycling infrastructure and pollution exposure attributes, particularly for maximum exposure levels.

## Introduction

### Population homogeneity

Discrete choice models and their variants are employed extensively for analyzing decision processes in various fields including transportation, marketing, social science, bio-statistics, and epidemiology. In discrete choice models, decision maker’s choice behavior is examined as a response to several exogenous variables that include attributes of the choice alternative or characteristics of the decision maker. The widely employed traditional discrete choice models restrict the impact of exogenous variables to be the same across the entire sample of records. The assumption is referred to as population homogeneity and is often highlighted as a limitation.

Several approaches have been employed to address population homogeneity restriction in discrete choice models. Segmenting the population based on exogenous variables and estimating separate models for each segment is a common approach. However, because there may be many variables to consider in the segmentation scheme, the number of segments (formed by the combination of the potential segmentation variables) can explode rapidly. To address the potential explosion of segments, clustering methods have been employed where target groups are divided into different clusters based on a multivariate set of factors and separate models are estimated for each cluster. However, both methods require allocating data records exclusively to a particular cluster, and do not consider the possible effects of unobserved factors that may moderate the impact of observed exogenous variables. Additionally, these approaches might result in very few records in some clusters resulting in loss of estimation efficiency.

A second approach to allow heterogeneity effects (variations in the effects of variables across the sample population) is to specify random coefficients (rather than imposing fixed coefficients) (for example, see [1–5]). But, while the mean of the random coefficients can be allowed to vary across decision makers based on observed exogenous variables, the random coefficients approach usually restricts the variance and the distributional form to be the same across all decision makers. A third approach to accommodate heterogeneity is to undertake an endogenous (or sometimes also referred to as latent) segmentation approach (see, for example [6–11]). In this approach, decision makers are allocated probabilistically to different segments, and segment-specific choice models are estimated. At the same time, each segment is identified based on a multivariate set of exogenous variables. The approach limits the number of segments to a manageable number (relative to the combinatorial scheme realized in the first approach).

A further extension of this approach would be accommodating unobserved heterogeneity within the segment specific choice models employing random parameters or error component model structures (see Hess and Stathopoulos [12]); thus subsuming the choice models from the second approach. Overall, the endogenous segmentation with segment level unobserved heterogeneity, offers an elegant alternative to address heterogeneity (observed and unobserved). In recent years, several studies have employed endogenous segmentation approaches (with or without unobserved heterogeneity) across different areas in transportation (for example, see [7–9, 11] in safety and see [6, 13–15] in travel behavior).

### Decision rule homogeneity

The exact formulation of discrete choice models are a function of the decision rule employed. In traditional discrete choice models, the analyst generally assumes the same decision rule across the sample population. The predominantly adopted decision rule for developing discrete choice models is random utility maximization (RUM) that hypothesizes that decision makers, when faced with multiple alternatives with varying attributes, choose the alternative that provides them with the highest utility or satisfaction [16–18]. While random utility model formulations have served as the predominant decision rule for discrete choice models, there is growing recognition of their limitations. The implicit compensatory nature of the formulation allows for a poor performance on an attribute (such as travel time) to be compensated by a positive performance on another attribute (such as travel cost) [19]. In some choice occasions, such behavior is not realistic. In recent years, motivated by research in behavioral economics, there has been considerable interest in alternative decision rules for discrete choice models such as relative advantage maximization [20], contextual concavity model [21], fully-compensatory decision making [22, 23], prospect theory (PT) [24, 25] and random regret minimization (RRM) [19, 26].

### Current study

Based on the aforementioned discussion, it is evident that homogeneity in both exogenous variable impact and decision rule restrict the flexibility offered by discrete choice models. In fact, the model parameters estimated with these restrictions are likely to be biased. While several research studies have focused on exogenous variable homogeneity, the decision rule homogeneity assumption has received less attention (for example see Hess et al. and Boeri et al. [27, 28]). The current research contributes to our understanding regarding heterogeneity in discrete choice models with respect to both exogenous variables and decision rules. Specifically, the proposed latent segmentation based mixed models segment population to different classes with their own decision rules while also incorporating unobserved heterogeneity within the segment level models. In our analysis, we choose to consider both random utility and random regret theories. The random regret minimization approach has received wide application because of its mathematical similarity to the random utility approach and its intuitive appeal [26, 29–34]. In Hess et al., [27] a two-segment latent class model is proposed–one segment represented by random utility formulation and the other by random regret formulation. In our approach, instead of assuming the number of segments (as 2), we conduct an exhaustive exploration with multiple segments across the two decision rules. Further, within each segment, we also allow for unobserved heterogeneity. The reader would note that the estimation of latent class models become complex with increasing number of segments and presence of unobserved heterogeneity (see Sobhani et al. [35] for some discussion). The extensive modeling exercise is developed employing a stated preference data compiled to understand influence of air pollution exposure on bicycle route choice.

The remainder of the paper is organized as follows. Next section provides a discussion of econometric methodology applied followed by the empirical context. In the section after, data source, variables considered, and model estimation results are presented in detail. Results from the trade-off analysis is presented in the fifth section. Final section presents a summary of findings and concludes the paper.

## Econometric framework

In this section, we describe the mathematical formulation of the model used in the current study. Let *c* (*c* = 1,2,…,*C*) be the index for cyclists, *i* (1,2,…,*I*) be the index for route alternatives characterized by *m* (*m* = 1,2,…,*M*) attributes, and *k* (1,2,…,*K*) be the index for choice occasions for each cyclist. In our case, *I* = 3 and *K* = 5 for all *c*. Let us also consider *S* possible number of segments where the cyclists would be probabilistically assigned. The probability that cyclist *c* belongs to segment *s* (*s* = 1,2,…,*S*) is given as:

$$P_{cs}=\frac{\exp\left(\gamma_{s}^{\prime }z_{c}\right)}{\sum_{s=1}^{S}\exp\left(\gamma_{s}^{\prime }z_{c}\right)}$$

*z*_*c*_ is a (M x 1) column vector of cyclist attributes that influences the propensity of belonging to segment *s*, $\gamma_{s}^{\prime }$ is a corresponding (M x 1) column vector of estimable coefficients. Within the latent class approach, the unconditional probability of a cyclist *c* choosing a commuting route *i* is given as:

$$P_{c}(i)=\sum_{s=1}^{S}(P_{c}(i)|s)(P_{cs})$$

where *P*_*c*_(*i*)|*s* represents the probability of cyclist *c* choosing route *i* within the segment *s*. Note that the decision paradigm used to obtain the conditional probability *P*_*c*_(*i*)|*s* may follow either utility or regret based unordered choice (traditionally multinomial logit) mechanism.

If a random utility based multinomial logit model is assumed to evaluate the route choice decision accommodating unobserved heterogeneity, the conditional probability would take the following form:

$$P_{c}(i)|s=\int \left(\prod_{k=1}^{K}\frac{\exp\left(\alpha_{s}^{\prime }x_{cik}\right)}{\sum_{r=1}^{R}\exp\left(\alpha_{s}^{\prime }x_{cik}\right))}\right)\;f(\alpha )d\alpha$$

Here, $\alpha_{s}^{\prime }$ is a (L x 1) column vector of coefficients, and *x*_*cik*_ is a (L x 1) column vector of route attributes, where *f*(*α*) is a density function specified to be normally distributed with mean 0 and variance *σ*^2^.

On the other hand, if a random regret based multinomial logit model is assumed to evaluate the route choice decision, the conditional probability would be given as:

$$P_{c}(i)|s=\int \left(\prod_{k=1}^{K}\frac{\exp\left(-R_{cik}\right)}{\sum_{r=1}^{R}\exp\left(-R_{cik}\right)}\right)\;f(\delta )d\delta$$

Here, $R_{cik}=\sum_{j\neq i}\sum_{m=1}^{M}\ln\left[1+exp\{\delta_{m}(x_{cjmk}-x_{cimk})\}\right]$; *δ*_*m*_ is a (Lx1) column vector of estimable coefficients associated with attribute *x*_*m*_; *x*_*im*_ and *x*_*jm*_ are (Lx1) column vectors of route attributes for the considered alternative *i* and another alternative *j*, respectively, where *f*(*δ*) is a density function specified to be normally distributed with mean 0 and variance *ρ*^2^. The log-likelihood function for the entire dataset with appropriate *P*_*c*_(*i*)|*s* is as follows:

$$LL=\sum_{c=1}^{C}log(P_{c}(i))$$

Contrary to the traditional endogenous segmentation approaches, capturing decision rule heterogeneity involves a more computationally intensive estimation approach. The estimation approach begins with single segment models from each regime. Then, a new segment from one of the two approaches is added. The process is continued until there is no further improvement in data fit. The approach allows for multiple segments originating from the same decision rule i.e. the segmentation model can have multiple RUM and RRM segments; thus offering enhanced flexibility. Finally, given the complexity of adding multiple segments from both regimes, we also consider overall sample shares of the segments in arriving at the final model as opposed to only data fit.

## Empirical context

The analysis of population and decision rule heterogeneity is conducted drawing on an empirical context–impact of air pollution on bicycle route choice. While bicycling offers health benefits, there is growing recognition that the potential health benefits might be offset by increased exposure to air pollutants for bicyclists. Several research efforts have documented the potential increased exposure to air pollution for bicyclists owing to their close proximity to traffic, high respiration rates, and longer journeys [36–38]. Furthermore, there is growing evidence from health research studies highlighting the potential consequences of increased air pollution exposure (for example see Weichenthal et al. [39]). Thus, there is need to explore the impact of air pollution exposure on bicycling choices.

An exhaustive review of literature on bicycling related decisions (such as decision to cycle, frequency of cycling, and route choice) is beyond the scope of the paper. Given the focus of our current study, we provide a concise summary of literature on route choice decision process for commuter cyclists (see Anowar et al. [40] for more details). For examining route choice, studies relied on both stated preference (SP) [41–48] and revealed preference (RP) survey data [49–53]. The most commonly employed analytical approaches to model route choice include binary logit (BL) or multinomial logit (MNL), mixed multinomial logit (MMNL), multinomial probit (MNP), and heuristic approaches. The important factors affecting route choice decision include socio-demographic characteristics, bike route characteristics, traffic characteristics, environmental attributes, access to facilities (such as showers at work place), and trip characteristics. Of these, the most significant factors are: travel time (lower is preferred), presence of incline (flat is preferred), bicycle infrastructure (continuous and exclusive/segregated routes are preferred), traffic volume (lower is preferred), and air pollution exposure (lower is preferred) [36, 40, 41, 43–47, 49, 50, 52, 54–56].

The current study builds on the first research effort that studied the impact of air pollution exposure on bicycling route choice (see Anowar et al., [40]). In the previous study, the emphasis was on examining if air pollution exposure information affected route choice. The study employed stated preference experiment data from 695 commuter cyclists and used a random utility approach to examine cyclist’s willingness to trade-off air pollution exposure with other attributes such as roadway characteristics, bike facilities, and travel time.

## Empirical analysis

### Data source and experimental design

In our SP survey, responses from bicyclists were collected along four dimensions. (1) Respondent’s personal and household characteristics (such as gender, age, education level, employment type and schedule, nearest intersections at the place of residence and work, household income, number of persons in the household, level of automobile and bicycle ownership, and commute time in minutes); (2) Cycling habits (frequency of cycling, if accompanied by children while making the trip, regular bicycling experience in years, primary reasons for cycling, seasons of cycling, and how often they switch their usual biking route); (3) Hypothetical choice scenarios with three route options per scenario; and (4) Cyclist’s perception about the characteristics of his/her usual commuting route.

The experimental design for identifying the hypothetical choice scenarios for the SP game was developed considering the following attributes: roadway characteristics: grade, traffic volume, and roadway type; bike route characteristics: cycling infrastructure continuity and segregation and landmarks along the route; and air pollution: mean exposure level (in ppb) and maximum exposure level (in ppb). A detailed description of the considered attributes and the corresponding attribute levels are presented in Table 1. Considering and comparing all of these attributes would burden the respondents significantly and complicate their route choice process. Hence, an innovative partitioning technique where only five attributes were used to characterize the alternative routes in each of the SP scenarios was used. Of these five attributes, the air pollution (mean and maximum exposure) and travel time attributes were always retained. These air pollution exposures were measured as a concentration of Nitrogen dioxide (NO_2_) in units of parts per billion (ppb). In addition, one attribute from roadway characteristics and one from bike route characteristics were randomly chosen for each individual through carefully designed rotating and overlapping approach to capture all variable effects when the responses from the different SP choice scenarios across different individuals are compiled together. Route choice alternatives were developed by experimental design routines in SAS in such a way that every individual gets five choice experiments in the survey. The SP scenarios were preceded by clear definitions of the attributes–pictorial representations were provided to give respondents a clearer idea about exclusive/shared and continuous/discontinuous cycling infrastructure.

**Table 1:** Attribute levels for the SP experiments.

Attribute Category	Attribute	Definition of Attribute	Attribute Levels
Roadway characteristics	Grade	Nature of terrain	1. Flat 2. Moderate 3. Steep
	Traffic volume	Amount of traffic on the roadway	1. Light 2. Moderate 3. Heavy
	Roadway type	Functional classification of roadway	1. Residential /Local roads 2. Minor arterial 3. Major arterial
Bike route characteristics	Cycling infrastructure continuity	Continuous bike route–if the whole route has a bicycle facility (a bike lane or shared-use path) Discontinuous—otherwise	1. Continuous 2. Discontinuous
	Cycling infrastructure segregation	Exclusive/Segregated–if physically separated from motor vehicle traffic Shared–otherwise	1. Exclusive 2. Shared
Environmental condition	Amount of traffic-related air pollution subjected to while cycling	Mean exposure levels to pollutants	1. 5 ppb 2. 10 ppb 3. 15 ppb
		Maximum exposure levels to pollutants	1. 20 ppb 2. 40 ppb 3. 60 ppb
Trip characteristics	Duration of trip	Travel time to destination (for commuting bicyclists only)	1. 20 minutes 2. 25 minutes 3. 30 minutes 4. 35 minutes 5. 40 minutes

We also conducted an “information provision” experiment to understand two issues. First, to identify if receiving information on the potential health effects resulting from exposure to traffic-related air pollution has any impact on a cyclist’s route choice decision and second, to study the sensitivity towards the nature of information provided. For this purpose, we devised three types of informational messages (see Supplementary information S1 Table for the messages). One (or none) of these messages was presented to the respondent in a window preceding the scenarios and following the description of attributes. The survey was designed so that information display was randomized to ensure that a quarter of the respondents received no information while the rest of them received at least one of the three messages. The details of the experimental design, attribute selection process, and survey dissemination strategies with demographic profile of commuters are described in Anowar et al. [40, 57].

The web-based survey was approved by the Health Sciences Research Ethics Board (HSREB) of the University of Toronto (UofT), Canada and was run from April 2016 through July 2016 for about 12 weeks. Several dissemination schemes were adopted including emailing web-link to the survey to individuals, university (University of Toronto and University of Central Florida) electronic mailing lists, various bicyclist forums, organizations, and groups; uploading posts in different social media platforms including Facebook, LinkedIn, and Twitter; placing advertisement posters in public message sharing spaces alongside major roadways (in Toronto). Additionally, bicycle-related websites posted the link on their web pages. Individuals who learnt about our survey from these sources may have distributed it to their peers, colleagues, family, and friends. Participation was completely voluntary and open to individuals over 18 years of age. At the beginning of the survey, participants were provided with an overview of what the survey entails and what it is for. They were given the option to proceed (I agree) or exit (I do not agree) from the survey, after reading the information. A total of 750 cyclists responded, out which 695 cyclists completed the survey.

### Data compilation and sample demographics

**Fig 1:**
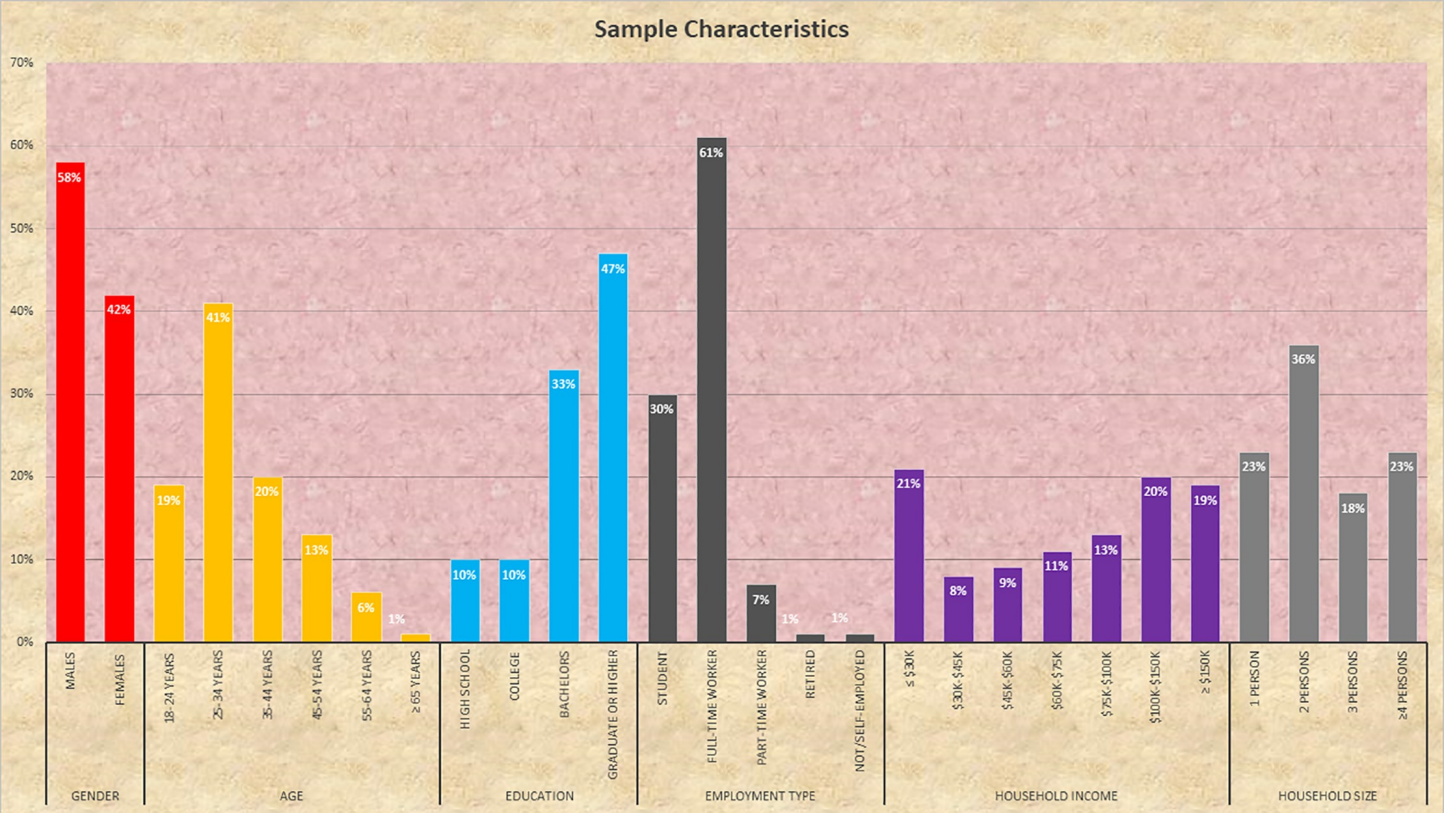
Socio-demographic profile of commuter cyclists.

The survey data was processed by removing incomplete information from raw data. A total of 3475 choices were compiled from 695 respondents. Fig 1 presents the descriptive statistics for the 695 commuter respondents from the sample. The sample of respondents is composed of 58 percent male and 42 percent female cyclists. Almost three-fifths (60%) of the respondents are aged between 18–34 years, reflecting that young adults are more likely to bicycle for commute purposes than older people. Almost fifty percent of commuter cyclists holds a graduate degree while almost three-fifths of cyclists are full-time job holders. About 40% of the commuter cyclists belong to a high-income household (more than $100,000/year). The majority (77%) of commuter cyclists reside in multi-individual households. A vast majority of them come from households owning multiple bicycles (77% of respondents’ household own at least 2 bicycles) while 42% of the respondents come from vehicle-less household. The reader would note that the survey participants include a higher proportion of younger, highly educated and high income households. While the sample is not representative of the general population, given that the emphasis is on route choice decision process, the lack of representativeness does not adversely affect the sample quality (see TCRP [58] and Sener et al. [46] for more discussion).

### Variables considered

In our study, we considered household and individual socio-demographic characteristics for latent segmentation component and bicycle route choice attributes for within segment models. The socio-demographic characteristics considered are: gender, age category, education, employment status, experience of bicycling, bicycling frequency, accompaniment by children, and actual commute time reported by respondents, number of household members, number of automobiles and bicycles owned by household. The variables considered for the route choice part are: (1) roadway characteristics: grade (flat, moderate, and steep), traffic volume (low, medium, and heavy), and roadway type (residential/local street, minor arterial, and major arterial), (2) bike route characteristics: cycling infrastructure continuity and cycling infrastructure segregation (exclusive and shared), and (3) air pollution (mean exposure level and maximum exposure level), and (4) trip characteristics: travel time.

Note that residential/local streets are those with light traffic with speeds < 40 km/h or 25 mph, minor arterials are those with moderate traffic with speeds 40–60 km/h or 25–40 mph, and major arterials are those with heavy traffic with speeds > 60 km/h or 40 mph. A bicycle route is labeled continuous if the whole route has a bicycle facility (a bike lane or a shared-use path). In contrast, a bicycle route is considered to be discontinuous if on some portions of the route bicyclists must share a lane with automobiles. Finally, exposure to traffic-generated pollution was expressed in two ways. First, mean exposure ranging from 5–15 ppb and maximum exposure ranging from 20–60 ppb. We used discretized travel time attribute ranging from 20–40 minutes.

### Model specification and performance evaluation

The empirical analysis involves estimation of several models. More specifically, we estimated four traditional models and nine latent class models. Four traditional models include: (1) random utility based multinomial logit model, (2) random utility based mixed multinomial logit model, (3) random regret based multinomial logit model, (4) random regret based mixed multinomial logit model. The estimated latent class models are: (1) random utility based latent multinomial logit model with two segments, (2) random regret based latent multinomial logit model with two segments, (3) random regret based latent multinomial logit model with three segments, (4) latent class multinomial logit model with hybrid segments (LCMHS). In the LCMHS category, we tested different combinations of decision rules with different number of classes. These are: (1) LCMHS with two segments (1 random utility based segment, 1 random regret based segment), (2) LCMHS with three segments (2 random regret based segment– 1 random utility based segment), (3) LCMHS with three segments (1 random regret based segment– 2 random utility based segment), (4) LCMHS with four segments (2 random regret based segment– 2 random regret based segment), (5) LCMHS with four segments (3 random regret based segment– 1 random utility based segment) and (6) LCMHS with four segments (1 random regret based segment– 3 random utility based segment). Note that we also tested for taste heterogeneity in the segment specific models, but the results were not supportive of the presence of further segment level unobserved heterogeneity. The variables that offered a statistically significant parameter at the 90% confidence level and offered intuitive impacts were retained.

The performance of the estimated (13) models was compared based on two goodness of fit measures best suited for comparing non-nested models: (1) Akaike information criterion (AIC) and (2) Bayesian Information Criterion (BIC). AIC for a given empirical model is expressed as:

AIC=2k−2ln(L)

where *k* is the estimated number of parameters and *L* denotes the maximized value of likelihood function for a given empirical model. The empirical equation of BIC is:

BIC=−2ln(L)+Kln(Q)

where *ln*(*L*) denotes the log likelihood value at convergence, *K* denotes the number of parameters, and *Q* represents the number of observations. Many of the earlier studies suggested that the BIC is the most consistent information criterion (IC) among all other traditionally used ICs (AIC, AICc, adjusted BIC) for number of segments selection in latent class models [6, 7, 11, 13, 59, 60]. The advantage of using BIC is that it imposes substantially higher penalty than other ICs on over-fitting. The model with the lowest AIC and BIC value is the preferred model. The BIC and AIC values for the final specifications of all the models are presented in Table 2. Based on these values, LCMHS with four segments (3 random regret based segment– 1 random utility based segment) offers the best data fit.

**Table 2:** Goodness of fit measures.

Model	Log-likelihood	Number of Parameters (K)	Number of Observations (Q)	BIC	AIC
**Traditional Choice Models**					
RUM based MNL	-2765.470	23	3475	5718.467	5576.940
RUM based mixed MNL	-2759.650	24	3475	5714.980	5567.300
RRM based MNL	-2709.500	35	3475	5704.367	5489.000
RRM based mixed MNL	-2688.781	32	3475	5638.470	5441.563
**Latent Segmentation Models**					
RUM based Latent MNL with two segments	-2734.217	20	3475	5631.500	5508.434
RRM based Latent MNL with two segments	-2693.295	23	3475	5574.118	5432.591
RRM based Latent MNL with three segments	-2665.158	26	3475	5542.304	5382.316
LCMS with two segments (1 RUM based segment-1 RRM based segment)	-2729.685	20	3475	5622.438	5499.371
LCMS with three segments (2 RUM based segment-1 RRM based segment)	-2601.792	36	3475	5497.104	5275.583
LCMS with three segments (1 RUM based segment-2 RRM based segment)	-2647.804	29	3475	5532.055	5353.608
LCMS with four segments (2 RUM based segment-2 RRM based segment)	-2559.369	42	3475	5461.178	5202.738
LCMS with four segments (1 RUM based segment-3 RRM based segment)	-2566.263	33	3475	**5401.587**	**5198.526**
LCMS with four segments (3 RUM based segment-1 RRM based segment)	-2624.438	34	3475	5526.090	5316.876

### Population share distribution among segments

The latent segmentation component determines the probability that a cyclist is assigned to the identified segments. We used the model estimations to generate the population shares across the various segments of all the latent class models following the equation [6, 61] below:

$$G_{S}=\frac{\sum_{c}P_{cs}}{C}$$

where *C* denotes the total number of respondents in the sample. The shares are presented in Table 3. The table offers some interesting insights. In all the latent class models with mixed choice paradigms, cyclists are more likely to be part of the segment(s) with random regret decision rule. For instance, in our best specified model, only 30% of the cyclists are likely to be allocated to the random utility based segment while the rest of them to the three random regret based segments (8%, 43%, and 19%). It is interesting to note that the split of cyclists who make their route choice decision following regret minimization concept is not equal.

**Table 3:** Population share distribution.

Model	Segment-1	Segment-2	Segment-3	Segment-4
RUM based Latent MNL with two segments	72	28	-	-
RRM based Latent MNL with two segments	47	53	-	-
LCMHS with two segments (1 RUM based segment-1 RRM based segment)	35	65	-	-
RRM based Latent MNL with three segments	16	18	66	-
LCMHS with three segments (2 RUM based segment-1 RRM based segment)	30	34	36	-
LCMHS with three segments (1 RUM based segment-2 RRM based segment)	24	21	55	-
LCMHS with four segments (2 RUM based segment-2 RRM based segment)	19	14	21	46
**LCMHS with four segments (1 RUM based segment-3 RRM based segment)**	**8**	**30**	**43**	**19**
LCMHS with four segments (3 RUM based segment-1 RRM based segment)	13	25	33	29

### Model results

In addition to the best model fit, LCMHS with four segments (3 random regret based segment– 1 random utility based segment) provided the most intuitive behavioral interpretation in terms of route choice decision. Hence, in this section we only discuss about the results of the best fit model in detail. Table 4 presents the results for the segmentation component (top panel of results) and segment specific route choice models (bottom panel of results). To provide a benchmark for the proposed model, we have also included the results for the mixed MNL model in Table 5.

**Table 4:** Results of LCMS with four segments (1 RUM based segment-3 RRM based segment).

Variables		Segment-1 (RRM)		Segment-2 (RUM)		Segment-3 (RRM)		Segment-4 (RRM)	
		Estimate	*t*-statistics	Estimate	*t*-statistics	Estimate	*t*-statistics	Estimate	*t*-statistics
**Segmentation Component**									
Constant		-	-	0.892	3.225	2.710	6.854	0.710	1.836
Female (Base: Male)		-	-	0.869	3.697	-	-	-	-
Age (Base: 18–34 years)									
	35 or more years	-	-	-	-	-1.119	-4.883	-	-
Auto Ownership		-	-	-	-	-0.498	-3.913	-	-
Biking frequency (Base: Rarely)									
	Daily	-	-	-	-	0.546	2.023	0.795	2.36
Commute length (Base: Short commute)									
	Long Commute	-	-	-	-	-1.013	-2.442	-	-
	Moderate to Long Commute	-	-	-	-	-	-	-0.978	-3.448
**Route Choice Component**									
**Roadway Characteristics**									
Grade (Base: Flat)									
	Steep	-	-	-1.795	-6.221	-2.131	-10.220	-	-
Traffic Volume (Base: Light)									
	Medium	-	-	-1.027	-3.492	-	-	-	-
	Heavy	-	-	-1.604	-5.906	-1.137	-6.399	-1.906	-5.760
Roadway Type (Base: Residential roads)									
	Minor arterial	-	-	-0.904	-5.156	-	-	-	-
	Major arterial	-	-	-2.178	-6.356	-1.843	-11.443	-	-
**Bike Route Characteristics**									
Infrastructure Continuity (Base: Discontinuous)									
	Continuous	-	-	1.325	6.071	1.000	5.486	-	-
Infrastructure Segregation (Base: Shared)									
	Exclusive	-	-	1.859	8.215	1.029	8.136	-	-
**Environmental condition**									
Mean Exposure		-0.055	-3.433	-0.058	-3.027	-0.067	-5.776	-0.050	-3.404
Maximum Exposure		-	-	-0.034	-6.957	-0.015	-5.723	-0.027	-6.984
**Trip Characteristics**									
Travel Time		-	-	-0.050	-4.247	-0.248	-12.122	-0.139	-8.205
Log-likelihood at Convergence		-2566.263							

**Table 5:** Results of RUM based mixed MNL.

Attribute Category	Attribute	Attribute Levels			Coefficient	*t*-statistics
Roadway Characteristics	Grade (Base: Flat)	Steep			-0.982	-10.579
			Female		-0.804	-5.601
	Traffic Volume (Base: Light)	Moderate			-0.657	-7.729
		Heavy			-1.508	-16.662
	Roadway Type (Base: Residential Roads)	Minor arterial			-0.398	-4.776
		Major arterial			-1.290	-15.025
			Female		-0.345	-2.576
Bike Route Characteristics	Infrastructure Continuity (Base: Discontinuous)	Continuous			0.879	13.485
	Infrastructure Segregation (Base: Shared)	Exclusive			0.939	10.353
			Female		0.306	2.561
Environmental Condition	Mean Exposure	Mean exposure			-0.054	-8.791
			Biking experience (Base: 2 or more years)			
				Less than 2 years	-0.021	-1.961
	Maximum Exposure	Maximum exposure			-0.019	-10.271
		*Standard deviation*			0.016	6.480
			Exposure impact information (Base: No information)			
				Short-term	-0.007	-2.148
Trip Characteristics	Travel Time	Travel time			-0.075	-4.551
			Female		0.018	2.942
			Age (Base: 18–24 years)			
				25–34 years	-0.043	-6.740
				55–64 years	0.027	2.656
				65 years or more	0.056	2.762
			Biking frequency (Base: Rarely)			
				Once or several times a month	-0.049	-2.988
				Daily	-0.080	-4.982
			Commute length (Base: Short commute)			
				Moderate	0.030	4.831
				Long	0.072	7.997
Log-likelihood at convergence (N = 3475): -2759.650						

#### Latent segmentation component

The variables in the segmentation part with positive (negative) coefficient indicate increase (decrease) in the propensity of the cyclists being part of the segment. In our analysis, we considered Segment 1 as the base. The positive sign on the constant term does not have any functional interpretation, but simply reflects the larger likelihood of bicyclists being part of other three segments. The variables influencing segment membership include gender, age, auto ownership, biking frequency, and commute length. Our results indicate that female bicyclists are more likely to be assigned to Segment 2 (utility based decision rule segment). Examining the coefficients of Segment 3, we find that bicyclists in this class are more likely to be daily commuters, less than 35 years of age, from a household with less number of automobiles, and have a moderate commute duration. Interestingly, Segment 4 is more likely to be comprised of daily commuters as well (with a slightly higher propensity for Segment 4 membership than Segment 3 membership) but with short commute length.

#### Segment specific route choice models

A cursory examination of the results indicates the presence of the higher number of segment specific effects for Segment 2 and Segment 3. On the other hand, Segment 1 route choice behavior is only influenced by one variable. It is also evident that across the various segments, the variable impacts are significantly different manifesting the presence of population heterogeneity. We provide a discussion of model results across the 4 segments in this section by variable characteristics.

**Roadway characteristics.** Grade, traffic volume, and roadway type variables influence route choice behavior in segments 2, 3 and 4. As expected, for commuting purposes, steep roadway grades reduce the likelihood of choosing the route in both utility (Segment 2) and regret (Segment 3) segments. In Segment 2, the coefficient indicates a reduction in utility for routes with steep grade. In Segment 3, commuter bicyclists will be predisposed to lower regret toward routes with flat or moderate grades relative to routes with steep grades. Cyclists are inclined to avoid steep grade presumably because of the discomfort from rigorous physical activity while commuting to work (see similar results in Sener et al. and Anowar et al. [40, 46]). High vehicular traffic volume (medium and heavy) on roadway deters cyclists from choosing the route. In Segment 2, in particular, there is a larger drop in utility for routes with heavy traffic. The negative coefficients for heavy traffic volume in Segment 3 and Segment 4 suggest that regret reduces if traffic volume on the non-chosen alternatives is higher, thus reducing the likelihood for opting for route with heavy traffic (see similar result in Dill and Voros [62]). The presence of increased vehicular traffic will increase the probability of conflict between cyclists with motorized vehicles; so it is expected that commuter cyclists prefer routes with lower traffic levels. In terms of roadway type, routes on minor and major arterials (relative to routes on residential roads) are less likely to be chosen for commuting purpose. The effect is more pronounced in Segment 2, the utility for a route drops significantly when that route is located on a major arterial. In segment 3, the coefficient for major arterial is negative indicating that the regret associated with not choosing a route along major arterial is lower (relative to other alternatives). The results are quite intuitive and could be attributed to cyclist’s perception of higher level of safety on residential streets.

**Bike route characteristics.** The effect of bike route characteristics is found significant only in Segment 2 and Segment 3 –these two classes captured respondents who are highly sensitive to cycling infrastructure. The routes with continuous or segregated facilities are associated with higher utility in segment 2 and lower regret in segment 3 increasing the inclination to choose routes with continuous or segregated facilities relative to routes without continuous or segregated facilities. The results indicate that cyclists prefer to ride on a route with continuous cycling facility or on an exclusive route segregated from vehicular traffic with a slightly higher preference for exclusive routes. The result is expected and is reported in earlier research as well (see similar results in [55, 62–67]). On the other hand, the bicycle infrastructure variables have no impact on segment 1 and 4.

**Air pollution.** Of the two air pollution attributes, only mean exposure was found to affect route choice behavior across all segments. This essentially implies that irrespective of the decision rule, cyclists in all segments are strongly sensitive to exposing themselves to air pollution while on road. As expected, increase in mean exposure for a route reduces the likelihood that a bicyclist chooses the alternative. On the other hand, maximum exposure affects route choice behavior in segments 2, 3 and 4. The influence of maximum exposure is also along expected lines–increase in maximum exposure along the route reduces the probability of choosing that route (see Anowar et al. [40] for similar results). The reader would note that between mean and maximum exposure, the influence of mean exposure is consistently larger than the influence of maximum exposure on a parts per billion basis. The higher negative coefficient for mean exposure level indicates that cyclists are more sensitive towards a constant level of pollution on a regular basis rather than instantaneous exposure to pollution.

**Trip characteristics.** For commuters, travel time is an important determinant of route choice. The variable influences route choice decision in segments 2, 3 and 4. An increase in travel time is associated with reduction in utility or increase in regret for the route with longer travel time. Thus, that route have a lower probability of being chosen. Several studies have highlighted the impact of travel time along the same lines (see, Anowar et al. [40], Sener et al. [46] and Stinson and Bhat [66]). It is however, quite interesting that for segment 1, travel time is not a factor. The results highlight the behavior of a small population group that is focused solely on reducing their exposure to air pollution. The discovery of their presence would not have been possible without the 4 segment latent class model developed in our study.

**Information provision.** We tested for the effect of information provision on route choice in the model specification. However, in our latent class model framework, the variables representing the message received by the cyclist did not offer any statistically significant impact. The result indicates that while the exposure impact information could have influenced the route choice decision process, the impact is not statistically significant in our study.

## Trade-off analysis

Using the outputs from the model, we computed the time-based trade-offs, i.e. how much (in minutes) bicyclists are willing to travel extra for using routes with better facilities or less traffic-generated pollution. This analysis gives us an insight on how the trade-off values are varying across different segments of cyclists. For Segment 2, the calculation is straightforward–dividing the coefficient value of each attribute by the coefficient value of travel time. However, Segment 3 and Segment 4 are random regret based classes. When all attributes in a model are evaluated using random regret decision rule, the calculation of trade-offs is done using the following equation (Chorus, [68]):

$$\frac{\sum_{j\neq i}-\beta_{t}/(1+1/exp[\beta_{t}(t_{j}-t_{i})])}{\sum_{j\neq i}-\beta_{r}/(1+1/exp[\beta_{r}(r_{j}-r_{i})])}$$

where *β*_*t*_ and *β*_*r*_ are the estimated coefficients for the two attributes for which we are calculating the trade-off. In our case, the *r*^*th*^ attribute is travel time and the *t*^*th*^ attribute represents the attribute for which the “willingness to travel extra” for a one-unit increase/decrease is being investigated. The results from the trade-off exercise (for main effects only) are presented in Table 6.

**Table 6:** Time based trade-offs.

Attribute	Attribute Levels	Travel Times (minutes)										
		Segment-2 (RUM)	Segment-3 (RRM)					Segment-4 (RRM)				
		20–40	20	25	30	35	40	20	25	30	35	40
Grade	Steep	35.90	46.22	13.95	7.68	5.30	4.19	-	-	-	-	-
Traffic Volume	Medium	20.54	-	-	-	-	-	-	-	-	-	-
	Heavy	32.08	20.89	6.31	3.47	2.39	1.89	34.04	18.23	11.94	8.88	7.24
Roadway type	Minor Arterial	18.08	-	-	-	-	-	-	-	-	-	-
	Major Arterial	43.56	38.61	11.65	6.42	4.43	3.50	-	-	-	-	-
Infrastructure Continuity	Continuous	26.50	3.26	0.99	0.54	0.37	0.30	-	-	-	-	-
Infrastructure Segregation	Exclusive	37.18	3.29	0.99	0.55	0.38	0.30	-	-	-	-	-
Environmental Condition	Mean Exposure (5 ppb)	5.80	3.07	0.93	0.51	0.35	0.28	2.09	1.12	0.73	0.55	0.44
	Mean Exposure (10 ppb)	11.60	8.13	2.45	1.35	0.93	0.74	5.13	2.75	1.80	1.34	1.09
	Mean Exposure (15 ppb)	17.40	15.17	4.58	2.52	1.74	1.38	9.11	4.88	3.20	2.38	1.94
	Maximum Exposure (20 ppb)	13.60	2.84	0.86	0.47	0.33	0.26	3.44	1.84	1.21	0.90	0.73
	Maximum Exposure (40 ppb)	27.20	7.28	2.20	1.21	0.83	0.66	11.08	5.93	3.88	2.89	2.36
	Maximum Exposure (60 ppb)	40.80	13.32	4.02	2.21	1.53	1.21	22.91	12.26	8.03	5.97	4.87

The results of the trade-off analysis provides some interesting insights. For the utility oriented segment, as expected, cyclists are willing to travel 15–45 minutes extra to avoid steep grade, medium/heavy traffic volume, and riding on routes along minor/major arterial. Moreover, they are also willing to travel in excess of 25 minutes to ride on a continuous or exclusive bike facility. “Value of Clean Ride (VCR)” for mean exposure, was estimated as 1.16 min/ppb and for maximum exposure, was estimated as 0.68 min/ppb suggesting that commuter cyclists are more sensitive to mean exposure than maximum exposure. The value obtained in our current analysis is double the value we obtained in our previous analysis (see [40]). This signifies that Segment 2 commuter cyclists, who more likely to be females, are strongly sensitive to air pollution and are willing to travel 5–40 minutes extra to avoid them.

Trade-off values from random utility paradigm is insensitive to the changes in the attribute values. However, we can see from Table 6 that random regret formulation based trade-offs calculated for Segment 3 and 4 are alternative and choice set dependent and monotonically decrease with increase in travel time. For example, from trade-off values, we can see that when a chosen alternative does poorly in terms of roadway attribute (has steep grade, or has heavy vehicular traffic or is located on a major arterial), but has a faster commuting time, an increase in travel time leads to a small increase in regret while improvement in terms of road grade leads to a relatively large decrease in regret. Hence, cyclists are willing to travel more than 40, 20, and 35 minutes, respectively for travelling on a route with better grades (medium or flat), better traffic situation (medium or low), and convenient roadway type (minor or residential). Cyclists in Segment 4 are willing to travel longer than cyclists in Segment 3 to avoid heavy traffic. Interestingly, the trade-off values in regret and utility based segments for roadway attributes are similar in magnitude; but values differ greatly for cycling infrastructure and exposure attributes, particularly for maximum exposure levels.

The Segment 3 and Segment 4 regret-based trade-off results might appear counter-intuitive on first glance. However, the reported results are a result of the construction of the RRM model. For alternatives with smaller travel times, any undesirable route feature (such as steep or high traffic volume) makes the alternative quite undesirable. Thus, individuals are willing to make larger trade-offs to avoid such features. The result is consistent across all attributes. At the lower end of travel time spectrum, the trade-off is quite high and drops as we move towards higher travel times. The result is analogous to the large shift in the “Value of Time (VoT)” values reported in Chorus [68]. Overall, these results clearly highlight how ignoring the presence of decision rule heterogeneity are likely to result in incorrect policy guidelines.

## Conclusions

In the extant literature, several approaches have been employed to address population homogeneity restriction in discrete choice models. Of these, latent class model is one of the elegant and intuitive approaches. Studies using latent class model have primarily focused on exogenous variable homogeneity; the decision rule homogeneity assumption has received less attention. Our study aims to bridge the gap in the literature in this context by analyzing population and decision rule heterogeneity simultaneously while drawing on a novel empirical context–impact of air pollution on bicycle route choice. In our analysis, we choose to consider the random utility framework along with random regret minimization approach. Further, instead of assuming the number of segments (as 2), we conduct an exhaustive exploration with multiple segments across the two decision rules. Within each segment we also allow for unobserved heterogeneity. The model estimation is conducted using a stated preference data from 695 commuter cyclists compiled through a web-based survey. Model fit measures revealed that latent class models with four segments (3 random regret based segment– 1 random utility based segment) provided the best data fit. The probabilistic allocation of respondents to different segments was achieved based on multivariate set of cyclist demographics and cycling habits. The results indicate that female commuter cyclists are more utility prone, however, the majority of the commuter cyclist’s choice pattern is consistent with regret minimization mechanism.

Overall, cyclists’ route choice decisions are influenced by roadway attributes, cycling infrastructure availability, pollution exposure, and travel time. Although travel time is the most important attribute for commuter cyclists in their route choice decision, it is however, quite interesting that for one of the segments, travel time is not a factor. The results highlight the behavior of a small population group that is focused solely on reducing their exposure to air pollution. The discovery of their presence would not have been possible without the 4 segment latent segmentation model developed in our study. This observation has interesting policy implications–it suggests that bicyclists’ exposure to air pollution should be incorporated in bicycle route planning. In addition, we find that between mean and maximum exposure, the influence of mean exposure is consistently larger than the influence of maximum exposure on a parts per billion basis. The higher negative coefficient for mean exposure level indicates that cyclists are more sensitive towards a constant level of pollution on a regular basis rather than instantaneous exposure to pollution. The analysis approach also allows us to investigate time based trade-offs across cyclists belonging to different classes. Interestingly, we observed that the trade-off values in regret and utility based segments for roadway attributes are similar in magnitude; but the values differ greatly for cycling infrastructure and exposure attributes, particularly for maximum exposure levels.

However, the study is not without limitations. The parameter estimates from our model systems are influenced by how respondents considered mean exposure and maximum exposure attributes. Given the scope of our survey, we could not educate bicyclists comprehensively on air quality measurement and impact of air quality on health. Our study is aimed to offer a guidance on how bicyclists respond to air quality information. Future research efforts can focus on offering additional approaches to providing air quality information in an effort to identify the most appropriate information dissemination framework.

## Supporting information

S1 Table Exposure impact information provision. (PDF)

S2 Table Results of RRM based mixed MNL. (PDF)

S3 Table Results of RUM based latent MNL with two segments. (PDF)

S4 Table Results of RRM Based latent MNL with two segments. (PDF)

S5 Table Results of LCMHS with two segments (1 RUM based segment-1 RRM based segment). (PDF)

S6 Table Results of RRM based latent MNL with three segments. (PDF)

S7 Table Results of LCMHS with three segments (1 RUM based segment-2 RRM based segment). (PDF)

S8 Table Results of LCMHS with three segments (2 RUM based segment-1 RRM based segment). (PDF)

S9 Table Results of LCMHS with four segments (2 RUM based segment-2 RRM based segment). (PDF)

S10 Table Results of LCMHS with four segments (3 RUM based segment-1 RRM based segment). (PDF)

S1 File Ethics approval. (PDF)

S2 File Survey questionnaire. (PDF)
