# Supplementary material for: Accommodating exogenous variable and decision rule heterogeneity in discrete choice models: Application to bicyclist route choice
Source: PLoS One. 2018 Nov 30;13(11):e0208309. doi: 10.1371/journal.pone.0208309 (PMC6268012; doi:10.1371/journal.pone.0208309)
Supplement: S1 Table — (PDF) [file pone.0208309.s001.pdf]

S1 Table. Exposure Impact Information Provision.

No message	No message
Short term exposure related message	Short term exposures to high levels of NO2 have been associated with immediate effects on heart rate (magnitude of the effect depends on the individual)
Long term exposure related message	Long term exposure to traffic related air pollution has been associated with a range of respiratory and cardiovascular health effects as well as some types of cancers
Specific information citing findings on long term exposure	Long-term exposure to traffic-related air pollution has been associated with a range of respiratory and cardiovascular health effects. Also, a recent study demonstrated that a 5 ppb increase in exposure to NO2 was associated with 10% increase in the risk of breast cancer. Another study also demonstrated that a 5ppb increase in exposure to NO2 was associated with 18% increase in the risk of prostate cancer
