# Supplementary material for: Accommodating exogenous variable and decision rule heterogeneity in discrete choice models: Application to bicyclist route choice
Source: PLoS One. 2018 Nov 30;13(11):e0208309. doi: 10.1371/journal.pone.0208309 (PMC6268012; doi:10.1371/journal.pone.0208309)
Supplement: S2 Table — (PDF) [file pone.0208309.s002.pdf]

S2 Table. Results of RRM Based mixed MNL.

Attribute Category	Attribute	Attribute Levels	Coefficient	*t*-statistics
Roadway Characteristics	Grade (Base: Flat)	Steep	-1.803	-3.897
		Female	-0.403	-3.916
		Age Range (Base: 18-24 Years)		
		25-34 Years	-0.596	-5.974
		Bicycling Experience (Base: More than 5 Years)		
		Less than 5 Years	-0.412	-3.910
		Accompanied (Base: With Children)		
		Without Children	1.033	2.653
	Traffic Volume (Base: Light)	Medium	-0.585	-5.607
		Age Range (Base: 18-24 Years)		
		45-54 Years	-0.395	-2.653
		Frequency of Bicycling (Base: Rarely)		
		Daily	0.301	2.197
		Heavy	-1.095	-18.011
	Roadway Type (Base: Residential Roads)	Minor Arterial	-0.245	-4.258
		Major Arterial	-0.667	-10.776
		Female	-0.221	-2.359
		Age Range (Base: 18-24 Years)		
		25-34 Years	-0.230	-2.408
Bike Route Characteristics	Infrastructure continuity (Base: Discontinuous)	Continuous	0.817	12.920
		Age Range (Base: Less than 35 Years)		
		35 Years or more	-0.242	-3.544
	Infrastructure segregation (Base: Shared)	Exclusive	0.826	8.604
		Female	0.229	2.520
		Frequency of Bicycling (Base: Rarely)		
		Daily	-0.196	-2.028
Environmental	Mean Exposure	Mean Exposure	-0.034	-5.858

Condition		Standard Deviation	0.069	11.331
		Bicycling Experience (Base: 2 or more Years)		
		Less than 2 Years	-0.027	-2.448
	Maximum Exposure	Maximum Exposure	-0.015	-11.136
		Standard Deviation	0.012	6.705
		Exposure impact information (Base: No information)		
		Short-term	-0.005	-2.298
Trip Characteristics	Travel Time	Travel time	-0.106	-16.615
		Female	0.017	3.675
		Age Range (Base: 18-24 Years)		
		25-34 Years	-0.033	-5.251
		35 Years or more	0.022	3.803
		Frequency of Bicycling (Base: Rarely)		
		Daily	-0.027	-5.842
		Bicycling Experience (Base: Less than 5 Years)		
		More than 5 Years	0.011	2.383
		Commute length (Base: Short commute)		
		Moderate	0.021	4.948
		Long	0.049	7.692
Log-likelihood at Convergence (N = 3475): -2688.781				
