# Supplementary material for: Accommodating exogenous variable and decision rule heterogeneity in discrete choice models: Application to bicyclist route choice
Source: PLoS One. 2018 Nov 30;13(11):e0208309. doi: 10.1371/journal.pone.0208309 (PMC6268012; doi:10.1371/journal.pone.0208309)
Supplement: S3 Table — (PDF) [file pone.0208309.s003.pdf]

S3 Table. Results of RUM Based Latent MNL With Two Segments.

Variables	Segment-1		Segment-2	
	**Estimate**	***t*-statistics**	**Estimate**	***t*-statistics**
**Segmentation Component**				
Constant	-	-	0.1207	0.544
Female (Base: Male)			-1.1213	-4.071
Age (Base: 18-34 years)				
35 or more years	-	-	-0.5829	-2.256
Biking frequency (Base: Rarely)				
Less than once to several times per month	-	-	-0.7634	-2.446
Commute length (Base: Short commute)				
Moderate to Long Commute	-	-	-0.5278	-2.103
**Route Choice Component**				
**Roadway Characteristics**				
Grade (Base: Flat)				
Steep	-1.9901	-10.796	-	-
Traffic Volume (Base: Light)				
Medium	-0.5979	-5.052	-	-
Heavy	-1.8195	-11.781	-	-
Roadway Type (Base: Residential roads)				
Minor arterial	-0.5826	-6.036	0.4712	2.619
Major arterial	-2.1185	-12.618	-	-
**Bike Route Characteristics**				
Infrastructure Continuity (Base: Discontinuous)				
Continuous	1.0168	10.244	-	-
Infrastructure Segregation (Base: Shared)				
Exclusive	1.4147	11.821	0.3814	2.126
**Environmental condition**				
Mean Exposure	-0.0646	-7.174	-0.0511	-3.118
Maximum Exposure	-0.0169	-8.678	-0.0341	-7.64
**Trip Characteristics**				
Travel Time	-0.1185	-18.196	-0.1816	-10.278
Log-likelihood at Convergence	-2734.216875			
