# Supplementary material for: Accommodating exogenous variable and decision rule heterogeneity in discrete choice models: Application to bicyclist route choice
Source: PLoS One. 2018 Nov 30;13(11):e0208309. doi: 10.1371/journal.pone.0208309 (PMC6268012; doi:10.1371/journal.pone.0208309)
Supplement: S4 Table — (PDF) [file pone.0208309.s004.pdf]

S4 Table. Results of RRM Based Latent MNL With Two Segments.

Variables	Segment-1		Segment-2	
	Estimate	*t*-statistics	Estimate	*t*-statistics
**Segmentation Component**				
Constant	-	-	0.5567	1.831
Age (Base: 18-34 years)				
35 or more years	-	-	-1.0154	-4.443
Auto Ownership				
2 or more	-	-	-0.7959	-2.835
Biking frequency (Base: Rarely)				
Daily	-	-	0.5885	2.319
Commute length (Base: Short commute)				
Moderate to Long Commute	-	-	-0.5905	-2.683
**Route Choice Component**				
**Roadway Characteristics**				
Grade (Base: Flat)				
Steep	-0.3618	-4.058	-1.7995	-10.907
Traffic Volume (Base: Light)				
Medium	-0.395	-3.653	-	-
Heavy	-0.8146	-7.267	-1.263	-9.178
Roadway Type (Base: Residential roads)				
Minor arterial	-0.4006	-5.628	-	-
Major arterial	-0.9872	-5.95	-0.8877	-5.647
**Bike Route Characteristics**				
Infrastructure Continuity (Base: Discontinuous)				
Continuous	0.5074	6	0.8509	6.739
Infrastructure Segregation (Base: Shared)				
Exclusive	0.6684	8.75	1.342	4.336
**Environmental condition**				
Mean Exposure	-0.0425	-6.243	-0.0524	-5.857
Maximum Exposure	-0.0165	-10.23	-0.0156	-7.7
**Trip Characteristics**				
Travel Time	-0.0354	-6.71	-0.2045	-13.681
Log-likelihood at Convergence	-2693.295275			
