# Supplementary material for: Accommodating exogenous variable and decision rule heterogeneity in discrete choice models: Application to bicyclist route choice
Source: PLoS One. 2018 Nov 30;13(11):e0208309. doi: 10.1371/journal.pone.0208309 (PMC6268012; doi:10.1371/journal.pone.0208309)
Supplement: S5 Table — (PDF) [file pone.0208309.s005.pdf]

S5 Table. Results of LCMHS With Two Segments (1 RUM Based Segment-1 RRM Based Segment).

Variables	Segment-1 (RRM)		Segment-2 (RUM)	
	Estimate	*t*-statistics	Estimate	*t*-statistics
**Segmentation Component**				
Constant	-	-	1.0009	3.041
Female (Base: Male)	-	-	0.4835	2.03
Age (Base: 18-34 years)				
35 or more years	-	-	-0.5952	-2.294
Auto Ownership	-	-	-0.3474	-2.777
Income (Base: Low Income)				
High Income	-	-	0.638	2.555
**Route Choice Component**				
**Roadway Characteristics**				
Grade (Base: Flat)				
Steep	-0.414	-3.783	-2.3841	-8.804
Traffic Volume (Base: Light)				
Medium	-	-	-1.2563	-7.065
Heavy	-	-	-2.4797	-11.709
Roadway Type (Base: Residential roads)				
Minor arterial	-	-	-0.6397	-5.518
Major arterial	-	-	-2.8088	-12.347
**Bike Route Characteristics**				
Infrastructure Continuity (Base: Discontinuous)				
Continuous	-	-	1.353	8.485
Infrastructure Segregation (Base: Shared)				
Exclusive	0.4319	3.962	1.484	10.007
**Environmental condition**				
Mean Exposure	-0.0401	-4.813	-0.0475	-4.069
Maximum Exposure	-0.0187	-8.432	-0.0144	-4.71
**Trip Characteristics**				
Travel Time	-0.0442	-7.943	-0.2009	-14.887
Log-likelihood at Convergence	-2729.685475			
