# Supplementary material for: Accommodating exogenous variable and decision rule heterogeneity in discrete choice models: Application to bicyclist route choice
Source: PLoS One. 2018 Nov 30;13(11):e0208309. doi: 10.1371/journal.pone.0208309 (PMC6268012; doi:10.1371/journal.pone.0208309)
Supplement: S6 Table — (PDF) [file pone.0208309.s006.pdf]

S6 Table. Results of RRM Based Latent MNL With Three Segments.

Variables	Segment-1		Segment-2		Segment-3	
	Estimate	*t*-statistics	Estimate	*t*-statistics	Estimate	*t*-statistics
**Segmentation Component**						
Constant	-	-	-0.2721	-0.94	2.2434	5.432
Female (Base: Male)	-	-	0.8565	3.026	-	-
Age (Base: 18-34 years)						
35 or more years	-	-	-	-	-0.8104	-3.45
Auto Ownership	-	-	-	-	-0.4917	-3.843
Biking frequency (Base: Rarely)						
Daily	-	-	-	-	0.8847	3.518
Biking experience (Base: Less than 2 years)						
2 to 5 Years	-	-	-	-	0.9802	2.95
Commute length (Base: Short commute)						
Moderate to Long Commute	-	-	-	-	-0.6445	-2.747
**Route Choice Component**						
**Roadway Characteristics**						
Grade (Base: Flat)						
Steep	-	-	-1.4436	-4.439	-1.2822	-10.691
Traffic Volume (Base: Light)						
Heavy	-0.788	-3.684	-0.5474	-3.304	-1.0213	-11.128
Roadway Type (Base: Residential roads)						
Minor arterial	-	-	-0.4583	-2.085	-	-
Major arterial	-	-	-	-	-1.2264	-13.715
**Bike Route Characteristics**						
Infrastructure Continuity (Base: Discontinuous)						
Continuous	-	-	1.5098	4.963	0.6078	7.015
Infrastructure Segregation (Base: Shared)						
Exclusive	-	-	2.1802	4.908	0.7595	9.181
**Environmental condition**						
Mean Exposure	-0.0407	-3.948	-	-	-0.0505	-7.328
Maximum Exposure	-0.0145	-5.767	-0.0199	-5.77	-0.016	-10.507
**Trip Characteristics**						
Travel Time	-0.0354	-6.71	-0.0281	-2.853	-0.1706	-17.948
Log-likelihood at Convergence	-2665.1582					
