# Supplementary material for: Accommodating exogenous variable and decision rule heterogeneity in discrete choice models: Application to bicyclist route choice
Source: PLoS One. 2018 Nov 30;13(11):e0208309. doi: 10.1371/journal.pone.0208309 (PMC6268012; doi:10.1371/journal.pone.0208309)
Supplement: S7 Table — (PDF) [file pone.0208309.s007.pdf]

S7 Table. Results of LCMHS With Three Segments (1 RUM Based Segment-2 RRM Based Segment).

Variables	Segment-1 (RRM)		Segment-2 (RUM)		Segment-3 (RRM)	
	Estimate	*t*-statistics	Estimate	*t*-statistics	Estimate	*t*-statistics
**Segmentation Component**						
Constant	-	-	-2.4359	-5.031	-0.0287	-0.107
Female (Base: Male)	-	-	-	-	0.4435	2.019
Age (Base: 18-34 years)						
35 or more years	-	-	1.0596	3.489	-	-
Auto Ownership	-	-	0.4901	3.429	-	-
Biking experience (Base:5 years or more)						
Less than 2 years	-	-	0.9852	2.508	-	-
Less than 5 years	-	-	-	-	0.6805	2.792
Commute length (Base: Short commute)						
Moderate to Long Commute	-	-	1.3112	3.775	0.9811	3.42
**Route Choice Component**						
**Roadway Characteristics**						
Grade (Base: Flat)						
Steep	-	-	-	-	-1.8319	-10.961
Traffic Volume (Base: Light)						
Medium	-0.835	-2.548	-	-	-	-
Heavy	-1.802	-5.638	-1.1947	-5.032	-0.7621	-7.02
Roadway Type (Base: Residential roads)						
Major arterial	-0.4642	-2.663	-	-	-1.7609	-10.852
**Bike Route Characteristics**						
Infrastructure Continuity (Base: Discontinuous)						
Continuous	0.4694	2.587	-	-	0.8338	7.614
Infrastructure Segregation (Base: Shared)						
Exclusive	0.4709	2.507	0.8839	5.572	1.1475	10.312
**Environmental condition**						
Mean Exposure	-0.0647	-3.689	-0.0457	-3.517	-0.0326	-3.902
Maximum Exposure	-0.0199	-5.884	-0.02	-5.962	-0.0176	-8.508
**Trip Characteristics**						
Travel Time	-0.1954	-8.568	-	-	-0.1385	-15.047
Log-likelihood at Convergence			-2647.80405			
