# Supplementary material for: Accommodating exogenous variable and decision rule heterogeneity in discrete choice models: Application to bicyclist route choice
Source: PLoS One. 2018 Nov 30;13(11):e0208309. doi: 10.1371/journal.pone.0208309 (PMC6268012; doi:10.1371/journal.pone.0208309)
Supplement: S8 Table — (PDF) [file pone.0208309.s008.pdf]

S8 Table. Results of LCMHS With Three Segments (2 RUM Based Segment-1 RRM Based Segment).

Variables	Segment-1 (RRM)		Segment-2 (RUM)		Segment-3 (RUM)	
	Estimate	*t*-statistics	Estimate	*t*-statistics	Estimate	*t*-statistics
**Segmentation Component**						
Constant	-	-	0.5664	1.616	-0.3167	-0.583
Female (Base: Male)	-	-	-	-	0.9036	3.871
Age (Base: 18-34 years)						
35 or more years	-	-	-0.855	-3.194	-	-
Employment Status						
Full-time or Part-time Worker	-	-	0.6878	2.417	0.72	2.492
Number of Household Member	-	-	-	-	0.3255	2.746
Bicycle Ownership	-	-	-	-	-0.4846	-3.008
Auto Ownership	-	-	-0.4639	-3.359	-	-
Biking experience (Base:5 years or more)						
Less than 5 years	-	-	0.5126	2.194	-	-
Commute length (Base: Short commute)						
Moderate to Long Commute	-	-	-	-	0.7794	3.375
**Route Choice Component**						
**Roadway Characteristics**						
Grade (Base: Flat)						
Steep	-0.3006	-2.325	-4.8934	-6.019	-1.6774	-6.893
Traffic Volume (Base: Light)						
Medium	-0.6115	-3.455	-	-	-0.4568	-2.495
Heavy	-1.1337	-7.485	-2.4846	-5.153	-1.0687	-5.916
Roadway Type (Base: Residential roads)						
Minor arterial	-	-	-0.8284	-3.376	-0.7873	-5.053
Major arterial	-	-	-4.4786	-9.179	-2.2092	-7.949
**Bike Route Characteristics**						
Infrastructure Continuity (Base: Discontinuous)						
Continuous	-	-	1.9149	4.124	0.9804	6.286
Infrastructure Segregation (Base: Shared)						
Exclusive	0.2954	2.552	1.5321	5.593	1.4442	8.488
**Environmental condition**						
Mean Exposure	-0.0653	-5.877	-0.0782	-3.535	-0.0365	-2.692
Maximum Exposure	-0.0248	-9.868	-	-	-0.019	-6.29
**Trip Characteristics**						

Travel Time	-0.0912	-11.681	-0.423	-8.299	-0.0417	-4.39
Log-likelihood at Convergence			-2601.791575			
