# Supplementary material for: Accommodating exogenous variable and decision rule heterogeneity in discrete choice models: Application to bicyclist route choice
Source: PLoS One. 2018 Nov 30;13(11):e0208309. doi: 10.1371/journal.pone.0208309 (PMC6268012; doi:10.1371/journal.pone.0208309)
Supplement: S9 Table — (PDF) [file pone.0208309.s009.pdf]

S9 Table. Results of LCMHS With Four Segments (2 RUM Based Segment-2 RRM Based Segment).

Variables	Segment-1 (RRM)		Segment-2 (RUM)		Segment-3 (RUM)		Segment-4 (RRM)	
	Estimate	*t*-statistics	Estimate	*t*-statistics	Estimate	*t*-statistics	Estimate	*t*-statistics
**Segmentation Component**								
Constant	-	-	-0.3441	-1.554	0.845	1.591	2.0661	5.166
Female (Base: Male)	-	-	-	-	-0.7824	-2.556	-	-
Age (Base: 18-34 years)								
35 or more years	-	-	-	-	-0.6656	-2.02	-1.2007	-4.733
Number of Household Member	-	-	-	-	-0.2769	-2.048	-	-
Auto Ownership	-	-	-	-	-	-	-0.5001	-3.862
Biking frequency (Base: Rarely)								
Daily	-	-	-	-	0.9291	2.46	0.5643	2.024
Commute length (Base: Short commute)								
Long Commute	-	-	-	-	-1.4334	-2.071	-1.1423	-2.723
**Route Choice Component**								
**Roadway Characteristics**								
Grade (Base: Flat)								
Steep	-0.9015	-5.908	-	-	-	-	-2.0274	-9.947
Traffic Volume (Base: Light)								
Medium	-0.6841	-3.859	1.0208	2.715	-1.3025	-2.372	-	-
Heavy	-1.0481	-5.383	-	-	-2.656	-3.154	-1.1102	-6.132
Roadway Type (Base: Residential roads)								
Minor arterial	-	-	-1.2814	-3.477	-	-	-0.2789	-2.263
Major arterial	-0.5335	-3.013	-1.8661	-4.236	-	-	-1.8005	-12.094
**Bike Route Characteristics**								
Infrastructure Continuity (Base: Discontinuous)								
Continuous	-	-	2.0755	6.536	0.6078	2.392	0.9639	5.703
Infrastructure Segregation (Base: Shared)								
Exclusive	0.3327	2.506	2.9195	4.47	0.7444	2.92	0.8961	6.778
**Environmental condition**								
Mean Exposure	-0.0398	-3.671	-	-	-0.1272	-4.429	-0.0463	-4.149
Maximum Exposure	-0.0228	-8.025	-0.0159	-2.254	-0.0319	-6.427	-0.0155	-6.167
**Trip Characteristics**								
Travel Time	-	-	-0.0489	-2.858	-0.2252	-8.513	-0.2004	-11.018
Log-likelihood at Convergence	-2559.368775							
