# Supplementary material for: Accommodating exogenous variable and decision rule heterogeneity in discrete choice models: Application to bicyclist route choice
Source: PLoS One. 2018 Nov 30;13(11):e0208309. doi: 10.1371/journal.pone.0208309 (PMC6268012; doi:10.1371/journal.pone.0208309)
Supplement: S10 Table — (PDF) [file pone.0208309.s010.pdf]

S10 Table. Results of LCMHS With Four Segments (3 RUM Based Segment-1 RRM Based Segment).

Variables	Segment-1 (RRM)		Segment-2 (RUM)		Segment-3 (RUM)		Segment-4 (RUM)	
	Estimate	*t*-statistics	Estimate	*t*-statistics	Estimate	*t*-statistics	Estimate	*t*-statistics
**Segmentation Component**								
Constant	-	-	-1.4278	-2.539	3.9151	3.571	0.9716	3.676
Female (Base: Male)	-	-	0.512	2.079	-	-	-	-
Age (Base: 18-34 years)								
35 or more years	-	-	-	-	-	-	-0.6226	-2.475
Employment Status								
Full-time or Part-time Worker	-	-	0.6862	2.301	-	-	-	-
Number of Household Member	-	-	1.2878	3.17	-	-	-	-
Bicycle Ownership (Less than 2)								
2 or more	-	-	-0.7503	-2.254	-	-	-	-
Auto Ownership	-	-	0.3698	2.623	-	-	-	-
Accompany (Base: With Children)								
No Children	-	-	-	-	-1.6005	-2.861	-	-
Commute length (Base: Short commute)								
Long Commute	-	-	1.5101	3.004	1.1199	2.121	-	-
**Route Choice Component**								
**Roadway Characteristics**								
Grade (Base: Flat)								
Steep	-	-	-	-	-3.132	-6.531	-5.0334	-7.418
Traffic Volume (Base: Light)								
Medium	-	-	-	-	-1.2787	-4.867	2.0723	2.057
Heavy	-	-	-0.7466	-3.913	-2.3774	-8.497		
Roadway Type (Base: Residential roads)								
Minor arterial	-	-	-0.754	-4.215	-	-	-1.0871	-3.113
Major arterial	-	-	-2.0681	-7.849	-	-	-6.8506	-8.125
**Bike Route Characteristics**								
Infrastructure Continuity (Base: Discontinuous)								
Continuous	-	-	0.9905	5.115	0.7945	3.588	-	-
Infrastructure Segregation (Base: Shared)								
Exclusive	-	-	1.4949	7.655	0.8669	4.293	1.8778	5.281
**Environmental condition**								
Mean Exposure	-	-	-	-	-0.121	-6.649	-	-
Maximum Exposure	-0.0393	-3.911	-	-	-0.0341	-6.655	-	-

**Trip Characteristics**								
Travel Time	-0.1386	-4.621	-0.0237	-2.229	-0.1401	-7.423	-0.4295	-9.089
Log-likelihood at Convergence	-2624.43815							
