# Supplementary material for: Accommodating exogenous variable and decision rule heterogeneity in discrete choice models: Application to bicyclist route choice
Source: PLoS One. 2018 Nov 30;13(11):e0208309. doi: 10.1371/journal.pone.0208309 (PMC6268012; doi:10.1371/journal.pone.0208309)
Supplement: S2 File — (PDF) [file pone.0208309.s012.pdf]

Impact of Air Pollution Information on Bicycle Route Choice: Commuter

Thank you in advance for taking the time to respond to the survey. Your participation is valuable to our research efforts.

We are conducting this survey to capture the impact of air pollution information on your route choice by bicycle.

Before proceeding, please read the consent form carefully.

No personal information will be collected in the survey (i.e. name and address). To ensure confidentiality of information, we will use a numeric code throughout this study. The results of this study may be published in scientific journals and communicated in other ways, but you will not be identifiable in any communications resulting from this study. All information will be stored on computers that are protected using passwords.

Your participation is voluntary. You have to be at least 18 years of age to take part in the survey. It will take you approximately 10-15 minutes to complete the survey. You can refuse to participate or can withdraw from this study at any time for any reason. If you click the "exit" button (at the top right hand corner of the survey screen), you will be allowed to exit from the survey and we will discard all of your responses. However, if you close the browser tab without clicking the exit button, your partial responses will be recorded. Refusal to participate or your withdrawal from the study will not involve any penalty.

If you have any questions and/or comments and/or would like to know about the study findings, feel free to contact: **Sabreena Anowar** (sabreena.anowar@utoronto.ca), Post-Doctoral Fellow, University of Toronto, Toronto, Canada; **Marianne Hatzopoulou** (marianne.hatzopoulou@utoronto.ca), Associate Professor, University of Toronto, Toronto, Canada; **Naveen Eluru** (naveen.eluru@ucf.edu), Associate Professor, University of Central Florida, Orlando, USA. You can also contact the Office of Research Ethics at ethics.review@utoronto.ca or 416-946-3273, if you have questions about your right as a participant.

The researchers have no conflicts of interest in this study.

* 1. Do you agree to the above terms? By clicking Yes, you consent that you are willing to answer the questions in this survey.

- ◯ Yes
- ◯ No

The survey has several sections.

Sections 1 and 2 cover questions about you and your cycling habits.

Section 3 presents a series of hypothetical scenarios and you are asked to state your preference.

Section 4 covers questions about your usual cycling route(s).

* 2. Which city do you currently reside in?

UNIVERSITY OF TORONTO University of Central Florida

Impact of Air Pollution Information on Bicycle Route Choice: Commuter

* 3. Are you?

◯ Female ◯ Male

* 4. What is your age?

◯ 18-24 ◯ 25-34 ◯ 35-44 ◯ 45-54 ◯ 55-64 ◯ Over 65

* 5. What is the highest level of education you have completed?

◯ High school ◯ College ◯ Bachelor’s ◯ Graduate or higher

* 6. What is your current working status ?

◯ Student ◯ Full-time worker ◯ Part-time worker ◯ Retired ◯ Not employed

UNIVERSITY OF TORONTO

University of Central Florida

Impact of Air Pollution Information on Bicycle Route Choice: Commuter

* 7. Is your work schedule?

◯ Flexible ◯ Rigid

* 8. Please provide the nearest cross-streets to your home

Cross-street 1: ____________________

Cross-street 2: ____________________

* 9. Please provide the nearest cross-streets to your work place

Cross-street 1: ____________________

Cross-street 2: ____________________

UNIVERSITY OF TORONTO University of Central Florida

* 10. Please provide the nearest cross-streets to your home

Cross-street 1:

Cross-street 2:

* 11. Please provide the nearest cross-streets to your school

Cross-street 1:

Cross-street 2:

Impact of Air Pollution Information on Bicycle Route Choice: Commuter

* 12. Please provide the nearest cross-streets to your home

Cross-street 1:

Cross-street 2:

UNIVERSITY OF TORONTO University of Central Florida

Impact of Air Pollution Information on Bicycle Route Choice: Commuter

* 13. What is your household's total annual income?

- ◯ Less than $30,000
- ◯ $30,001–$45,000
- ◯ $45,001–$60,000
- ◯ $60,001–$75,000
- ◯ $75,001–$100,000
- ◯ $100,001–$150,000
- ◯ More than $150,000

* 14. How many people are there in your household (including you)?

◯ 1 ◯ 2 ◯ 3 ◯ 4 or more

* 15. How many bicycles do your household own?

◯ 0 ◯ 1 ◯ 2 ◯ 3 or more

* 16. How many automobiles do your household own?

◯ 0 ◯ 1 ◯ 2 ◯ 3 or more

UNIVERSITY OF TORONTO

University of Central Florida

Impact of Air Pollution Information on Bicycle Route Choice: Commuter

* 17. How long (in minutes) is your usual commute (one way) using bicycle?

Impact of Air Pollution Information on Bicycle Route Choice: Commuter

* 18. How often do you bicycle?

- ◯ Rarely
- ◯ Less than once per month
- ◯ Several times a month
- ◯ Daily

* 19. Are you accompanied by children when you bicycle?

- ◯ Yes
- ◯ No

* 20. How long have you been regularly bicycling?

- ◯ More than 5 years
- ◯ Between 2-5 years
- ◯ Between 1-2 years
- ◯ Less than 1 year

* 21. What are your reasons for bicycling? (Check all that apply)

- ☐ Fitness/Health concerns
- ☐ Concern for environmental issues related to overuse of automobiles
- ☐ Accessibility/Convenience/Speed
- ☐ Avoid driving in congested conditions
- ☐ Avoid relying on public transit
- ☐ Pleasure/Enjoyment
- ☐ Due to limited auto parking at destinations
- ☐ Others (please specify)

Impact of Air Pollution Information on Bicycle Route Choice: Commuter

* 22. Please provide a rank **(1 for most important)** for each of your stated reasons for bicycling.

- Fitness/Health concerns
- Concern for environmental issues related to overuse of automobiles
- Accessibility/Convenience/Speed
- Avoid driving in congested conditions
- Avoid relying on public transit
- Pleasure/Enjoyment
- Due to limited auto parking at destinations
- [Insert text from Other]

* 23. During which time of year do you bicycle? (Check all that apply)

- [ ] May to August
- [ ] September to November
- [ ] December to February
- [ ] March to April

* 24. How many routes do you use for your usual commute?

○ I stick to the same route ○ I use 2-3 routes ○ I use more than 3 routes

A 25.0%

The next section presents a series of 5 hypothetical scenarios describing the characteristics of a set of bicycle routes and asks you to select your preferred route. Please take your time answering these questions - they are the most important part of the survey.

B 25.0%

Short term exposures to high levels of NO_2 have been associated with immediate effects on heart rate (magnitude of the effect depends on the individual)

The next section presents a series of 5 hypothetical scenarios describing the characteristics of a set of bicycle routes and asks you to select your preferred route. Please take your time answering these questions - they are the most important part of the survey.

C 25.0%

Long-term exposure to traffic-related air pollution has been associated with a range of respiratory and cardiovascular health effects as well as some types of cancers.

The next section presents a series of 5 hypothetical scenarios describing the characteristics of a set of bicycle routes and asks you to select your preferred route. Please take your time answering these questions - they are the most important part of the survey.

D 25.0%

Long-term exposure to traffic-related air pollution has been associated with a range of respiratory and cardiovascular health effects. Also, a recent study demonstrated that a 5ppb increase in exposure to nitrogen dioxide was associated with 10% increase in the risk of breast cancer. Another study also demonstrated that a 5ppb increase in exposure to nitrogen dioxide was associated with 18% increase in the risk of prostate cancer.

The next section presents a series of 5 hypothetical scenarios describing the characteristics of a set of bicycle routes and asks you to select your preferred route. Please take your time answering these questions - they are the most important part of the survey.

Impact of Air Pollution Information on Bicycle Route Choice: Commuter

Before you begin, please read the following definitions carefully

Attribute	Definition
Air pollution exposure	Amount of traffic-related air pollution subjected to while cycling. Air pollution exposure is measured as a concentration of Nitrogen dioxide (NO_2). This concentration is listed in units of parts per billion (ppb). NO_2 concentrations in cities like Toronto and Montreal in Canada typically range between 5ppb and 50ppb.<br><br>**Mean exposure** refers to the average level over the route.<br><br>The **maximum exposure** is the maximum level you would encounter for a short duration such as biking behind a bus/truck for part of your trip.<br><br>Both mean and maximum exposures matter to your health.

Before you begin, please read the following definitions carefully

Attribute	Definition
Roadway type	**Major arterial:** heavy traffic with speeds >60 km/h or 40mph<br><br>**Minor arterial:** moderate traffic with speeds 40-60 km/h or 25-40 mph<br><br>**Residential/local street:** light traffic with speeds <40 km/h or 25 mph

Impact of Air Pollution Information on Bicycle Route Choice: Commuter

Before you begin, please read the following definitions carefully

**Attribute**	**Definition**
Cycling infrastructure continuity	A bicycle route is considered to be **continuous** if the whole route has a bicycle facility (a bike lane or a shared-use path).<br><br>In contrast, a bicycle route is considered to be **discontinuous** if on some portions of the route bicyclists must share a lane with automobiles.

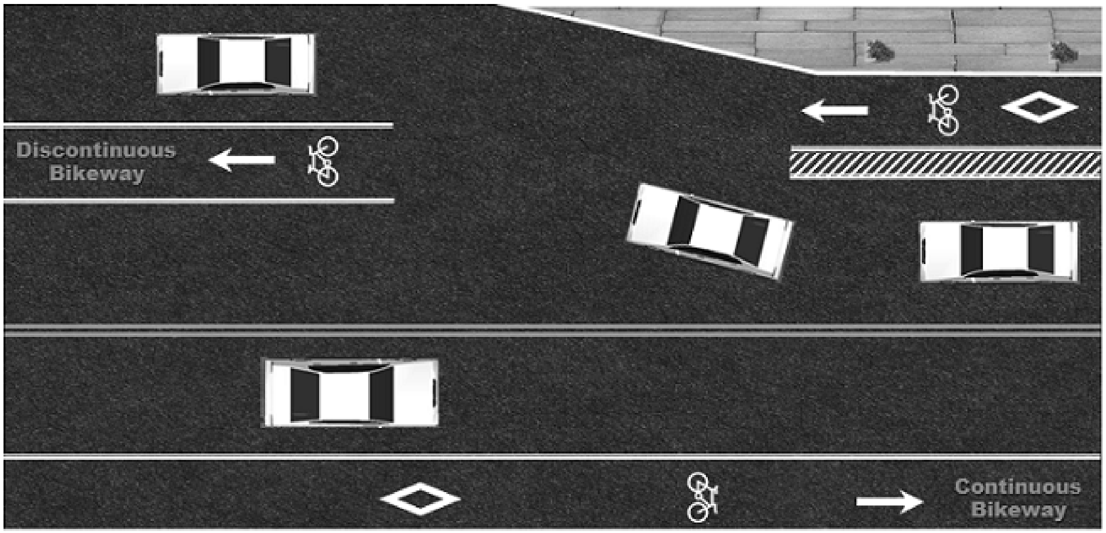

Impact of Air Pollution Information on Bicycle Route Choice: Commuter

Before you begin, please read the following definitions carefully

Attribute	Definition
Cycling infrastructure segregation	A bicycle route is considered to be **exclusive/segregated** if it is physically separated from motor vehicle traffic.<br><br>A bicycle route is considered to be **shared** if the route is not physically separated from motor vehicle traffic.

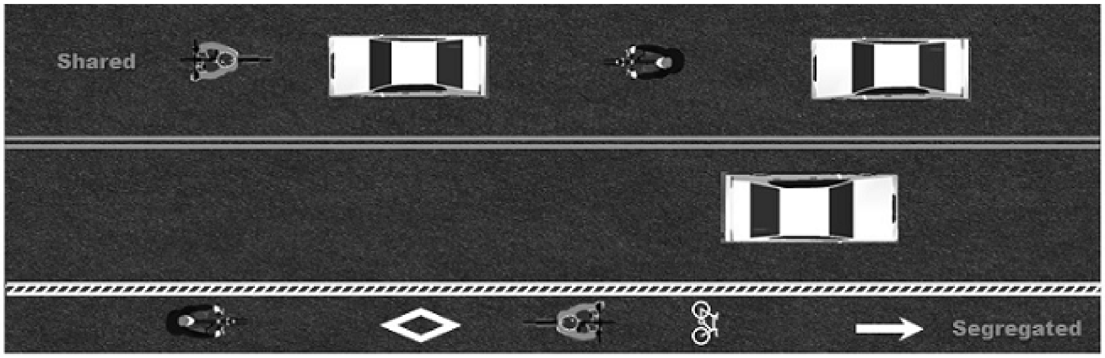

Impact of Air Pollution Information on Bicycle Route Choice: Commuter

Attribute	Route 1	Route 2	Route 3
Roadway grade	Flat	Steep	Moderate
Cycling infrastructure continuity	Continuous	Discontinuous	Discontinuous
Mean exposure level	15 ppb	5 ppb	10 ppb
Maximum exposure along the route	60 ppb	20 ppb	40 ppb
Travel time	20 minutes	30 minutes	25 minutes

* 25. Choose a route from these 3 alternatives.

◯ Route 1 ◯ Route 2 ◯ Route 3

Attribute	Route 1	Route 2	Route 3
Roadway grade	Moderate	Steep	Flat
Cycling infrastructure continuity	Continuous	Discontinuous	Discontinuous
Mean exposure level	15 ppb	10 ppb	5 ppb
Maximum exposure along the route	40 ppb	20 ppb	60 ppb
Travel time	30 minutes	25 minutes	35 minutes

* 26. Choose a route from these 3 alternatives.

◯ Route 1 ◯ Route 2 ◯ Route 3

Attribute	Route 1	Route 2	Route 3
Roadway grade	Steep	Moderate	Flat
Cycling infrastructure continuity	Discontinuous	Continuous	Discontinuous
Mean exposure level	10 ppb	5 ppb	15 ppb
Maximum exposure along the route	60 ppb	20 ppb	40 ppb
Travel time	20 minutes	25 minutes	35 minutes

* 27. Choose a route from these 3 alternatives.

◯ Route 1 ◯ Route 2 ◯ Route 3

Attribute	Route 1	Route 2	Route 3
Roadway grade	Steep	Moderate	Flat
Cycling infrastructure continuity	Discontinuous	Continuous	Continuous
Mean exposure level	5 ppb	15 ppb	10 ppb
Maximum exposure along the route	40 ppb	20 ppb	60 ppb
Travel time	25 minutes	40 minutes	30 minutes

* 28. Choose a route from these 3 alternatives.

◯ Route 1 ◯ Route 2 ◯ Route 3

Attribute	Route 1	Route 2	Route 3
Roadway grade	Steep	Moderate	Flat
Cycling infrastructure continuity	Continuous	Discontinuous	Continuous
Mean exposure level	5 ppb	10 ppb	15 ppb
Maximum exposure along the route	40 ppb	60 ppb	20 ppb
Travel time	40 minutes	25 minutes	20 minutes

* 29. Choose a route from these 3 alternatives.

◯ Route 1 ◯ Route 2 ◯ Route 3

Impact of Air Pollution Information on Bicycle Route Choice: Commuter

Attribute	Route 1	Route 2	Route 3
Roadway grade	Flat	Moderate	Steep
Cycling infrastructure segregation	Shared	Exclusive	Shared
Mean exposure level	5 ppb	15 ppb	10 ppb
Maximum exposure along the route	60 ppb	40 ppb	20 ppb
Travel time	40 minutes	25 minutes	35 minutes

* 30. Choose a route from these 3 alternatives.

◯ Route 1 ◯ Route 2 ◯ Route 3

Attribute	Route 1	Route 2	Route 3
Roadway grade	Steep	Moderate	Flat
Cycling infrastructure segregation	Exclusive	Shared	Shared
Mean exposure level	5 ppb	10 ppb	15 ppb
Maximum exposure along the route	40 ppb	20 ppb	60 ppb
Travel time	35 minutes	30 minutes	20 minutes

* 31. Choose a route from these 3 alternatives.

◯ Route 1 ◯ Route 2 ◯ Route 3

Attribute	Route 1	Route 2	Route 3
Roadway grade	Flat	Steep	Moderate
Cycling infrastructure segregation	Shared	Exclusive	Shared
Mean exposure level	15 ppb	10 ppb	5 ppb
Maximum exposure along the route	20 ppb	40 ppb	60 ppb
Travel time	30 minutes	20 minutes	35 minutes

* 32. Choose a route from these 3 alternatives.

◯ Route 1 ◯ Route 2 ◯ Route 3

Attribute	Route 1	Route 2	Route 3
Roadway grade	Flat	Steep	Moderate
Cycling infrastructure segregation	Exclusive	Shared	Exclusive
Mean exposure level	5 ppb	15 ppb	10 ppb
Maximum exposure along the route	60 ppb	20 ppb	40 ppb
Travel time	20 minutes	40 minutes	25 minutes

* 33. Choose a route from these 3 alternatives.

◯ Route 1 ◯ Route 2 ◯ Route 3

Attribute	Route 1	Route 2	Route 3
Roadway grade	Flat	Moderate	Steep
Cycling infrastructure segregation	Exclusive	Shared	Shared
Mean exposure level	5 ppb	15 ppb	10 ppb
Maximum exposure along the route	40 ppb	60 ppb	20 ppb
Travel time	40 minutes	35 minutes	20 minutes

* 34. Choose a route from these 3 alternatives.

◯ Route 1 ◯ Route 2 ◯ Route 3

Attribute	Route 1	Route 2	Route 3
Traffic volume	Heavy	Light	Moderate
Cycling infrastructure continuity	Continuous	Discontinuous	Continuous
Mean exposure level	15 ppb	5 ppb	10 ppb
Maximum exposure along the route	60 ppb	40 ppb	20 ppb
Travel time	20 minutes	30 minutes	40 minutes

* 35. Choose a route from these 3 alternatives.

◯ Route 1 ◯ Route 2 ◯ Route 3

Attribute	Route 1	Route 2	Route 3
Traffic volume	Heavy	Light	Moderate
Cycling infrastructure continuity	Continuous	Discontinuous	Continuous
Mean exposure level	15 ppb	5 ppb	10 ppb
Maximum exposure along the route	40 ppb	60 ppb	20 ppb
Travel time	20 minutes	25 minutes	35 minutes

* 36. Choose a route from these 3 alternatives.

◯ Route 1 ◯ Route 2 ◯ Route 3

Attribute	Route 1	Route 2	Route 3
Traffic volume	Heavy	Light	Moderate
Cycling infrastructure continuity	Continuous	Discontinuous	Continuous
Mean exposure level	5 ppb	15 ppb	10 ppb
Maximum exposure along the route	40 ppb	20 ppb	60 ppb
Travel time	30 minutes	25 minutes	40 minutes

* 37. Choose a route from these 3 alternatives.

◯ Route 1 ◯ Route 2 ◯ Route 3

Attribute	Route 1	Route 2	Route 3
Traffic volume	Moderate	Light	Heavy
Cycling infrastructure continuity	Discontinuous	Discontinuous	Continuous
Mean exposure level	5 ppb	15 ppb	10 ppb
Maximum exposure along the route	40 ppb	20 ppb	60 ppb
Travel time	40 minutes	25 minutes	30 minutes

* 38. Choose a route from these 3 alternatives.

◯ Route 1 ◯ Route 2 ◯ Route 3

Attribute	Route 1	Route 2	Route 3
Traffic volume	Heavy	Light	Moderate
Cycling infrastructure continuity	Continuous	Discontinuous	Continuous
Mean exposure level	15 ppb	10 ppb	5 ppb
Maximum exposure along the route	20 ppb	40 ppb	60 ppb
Travel time	35 minutes	20 minutes	40 minutes

* 39. Choose a route from these 3 alternatives.

◯ Route 1 ◯ Route 2 ◯ Route 3

Attribute	Route 1	Route 2	Route 3
Traffic volume	Light	Moderate	Heavy
Cycling infrastructure segregation	Exclusive	Shared	Exclusive
Mean exposure level	10 ppb	15 ppb	5 ppb
Maximum exposure along the route	40 ppb	60 ppb	20 ppb
Travel time	35 minutes	30 minutes	20 minutes

* 40. Choose a route from these 3 alternatives.

◯ Route 1 ◯ Route 2 ◯ Route 3

Attribute	Route 1	Route 2	Route 3
Traffic volume	Moderate	Light	Heavy
Cycling infrastructure segregation	Shared	Exclusive	Shared
Mean exposure level	5 ppb	15 ppb	10 ppb
Maximum exposure along the route	40 ppb	60 ppb	20 ppb
Travel time	20 minutes	40 minutes	30 minutes

* 41. Choose a route from these 3 alternatives.

◯ Route 1 ◯ Route 2 ◯ Route 3

Attribute	Route 1	Route 2	Route 3
Traffic volume	Light	Moderate	Heavy
Cycling infrastructure segregation	Exclusive	Exclusive	Shared
Mean exposure level	10 ppb	15 ppb	5 ppb
Maximum exposure along the route	40 ppb	60 ppb	20 ppb
Travel time	35 minutes	25 minutes	20 minutes

* 42. Choose a route from these 3 alternatives.

◯ Route 1 ◯ Route 2 ◯ Route 3

Attribute	Route 1	Route 2	Route 3
Traffic volume	Moderate	Light	Heavy
Cycling infrastructure segregation	Exclusive	Shared	Exclusive
Mean exposure level	15 ppb	5 ppb	10 ppb
Maximum exposure along the route	20 ppb	60 ppb	40 ppb
Travel time	20 minutes	25 minutes	35 minutes

* 43. Choose a route from these 3 alternatives.

◯ Route 1 ◯ Route 2 ◯ Route 3

Attribute	Route 1	Route 2	Route 3
Traffic volume	Heavy	Moderate	Light
Cycling infrastructure segregation	Exclusive	Shared	Exclusive
Mean exposure level	5 ppb	10 ppb	15 ppb
Maximum exposure along the route	40 ppb	60 ppb	20 ppb
Travel time	25 minutes	30 minutes	20 minutes

* 44. Choose a route from these 3 alternatives.

◯ Route 1 ◯ Route 2 ◯ Route 3

Impact of Air Pollution Information on Bicycle Route Choice: Commuter

Attribute	Route 1	Route 2	Route 3
Roadway type	Residential/local	Major	Minor
Cycling infrastructure continuity	Continuous	Discontinuous	Continuous
Mean exposure level	10 ppb	15 ppb	5 ppb
Maximum exposure along the route	40 ppb	20 ppb	60 ppb
Travel time	35 minutes	25 minutes	40 minutes

* 45. Choose a route from these 3 alternatives.

◯ Route 1 ◯ Route 2 ◯ Route 3

Attribute	Route 1	Route 2	Route 3
Roadway type	Residential/local	Major	Minor
Cycling infrastructure continuity	Continuous	Discontinuous	Discontinuous
Mean exposure level	15 ppb	10 ppb	5 ppb
Maximum exposure along the route	20 ppb	60 ppb	40 ppb
Travel time	40 minutes	25 minutes	30 minutes

* 46. Choose a route from these 3 alternatives.

◯ Route 1 ◯ Route 2 ◯ Route 3

Attribute	Route 1	Route 2	Route 3
Roadway type	Residential/local	Major	Minor
Cycling infrastructure continuity	Continuous	Discontinuous	Discontinuous
Mean exposure level	10 ppb	15 ppb	5 ppb
Maximum exposure along the route	60 ppb	20 ppb	40 ppb
Travel time	35 minutes	20 minutes	40 minutes

* 47. Choose a route from these 3 alternatives.

◯ Route 1 ◯ Route 2 ◯ Route 3

Attribute	Route 1	Route 2	Route 3
Roadway type	Minor	Major	Residential/local
Cycling infrastructure continuity	Discontinuous	Continuous	Discontinuous
Mean exposure level	15 ppb	5 ppb	10 ppb
Maximum exposure along the route	20 ppb	60 ppb	40 ppb
Travel time	30 minutes	40 minutes	35 minutes

* 48. Choose a route from these 3 alternatives.

◯ Route 1 ◯ Route 2 ◯ Route 3

Attribute	Route 1	Route 2	Route 3
Roadway type	Minor	Major	Residential/local
Cycling infrastructure continuity	Continuous	Discontinuous	Discontinuous
Mean exposure level	15 ppb	10 ppb	5 ppb
Maximum exposure along the route	20 ppb	60 ppb	40 ppb
Travel time	35 minutes	25 minutes	30 minutes

* 49. Choose a route from these 3 alternatives.

◯ Route 1 ◯ Route 2 ◯ Route 3

Impact of Air Pollution Information on Bicycle Route Choice: Commuter

Attribute	Route 1	Route 2	Route 3
Roadway type	Minor	Major	Residential/local
Cycling infrastructure segregation	Exclusive	Shared	Exclusive
Mean exposure level	15 ppb	5 ppb	10 ppb
Maximum exposure along the route	40 ppb	20 ppb	60 ppb
Travel time	40 minutes	35 minutes	30 minutes

* 50. Choose a route from these 3 alternatives.

◯ Route 1 ◯ Route 2 ◯ Route 3

Attribute	Route 1	Route 2	Route 3
Roadway type	Minor	Residential/local	Major
Cycling infrastructure segregation	Shared	Exclusive	Exclusive
Mean exposure level	15 ppb	10 ppb	5 ppb
Maximum exposure along the route	40 ppb	20 ppb	60 ppb
Travel time	35 minutes	40 minutes	30 minutes

* 51. Choose a route from these 3 alternatives.

◯ Route 1 ◯ Route 2 ◯ Route 3

Attribute	Route 1	Route 2	Route 3
Roadway type	Major	Minor	Residential/local
Cycling infrastructure segregation	Exclusive	Shared	Exclusive
Mean exposure level	5 ppb	10 ppb	15 ppb
Maximum exposure along the route	40 ppb	60 ppb	20 ppb
Travel time	40 minutes	20 minutes	25 minutes

* 52. Choose a route from these 3 alternatives.

◯ Route 1 ◯ Route 2 ◯ Route 3

Attribute	Route 1	Route 2	Route 3
Roadway type	Residential/local	Minor	Major
Cycling infrastructure segregation	Exclusive	Shared	Shared
Mean exposure level	15 ppb	5 ppb	10 ppb
Maximum exposure along the route	40 ppb	20 ppb	60 ppb
Travel time	30 minutes	40 minutes	25 minutes

* 53. Choose a route from these 3 alternatives.

◯ Route 1 ◯ Route 2 ◯ Route 3

Attribute	Route 1	Route 2	Route 3
Roadway type	Residential/local	Major	Minor
Cycling infrastructure segregation	Exclusive	Shared	Shared
Mean exposure level	15 ppb	10 ppb	5 ppb
Maximum exposure along the route	40 ppb	60 ppb	20 ppb
Travel time	30 minutes	20 minutes	35 minutes

* 54. Choose a route from these 3 alternatives.

◯ Route 1 ◯ Route 2 ◯ Route 3

You have almost reached the end of survey.

UNIVERSITY OF TORONTO

University of Central Florida

Impact of Air Pollution Information on Bicycle Route Choice: Commuter

* 55. Which of the following bicycle facilities exist on your usual route? (Check all that apply)

- [ ] Bicycle lane (a designated portion of the roadway striped for bicycle use)
- [ ] Unsigned shared roadway (a roadway without bike signage or movement marking)
- [ ] Signed shared roadway (a shared roadway designated by signing as a preferred route for bicycle use)
- [ ] Off-road bikeway (a bikeway physically separated from motorized vehicular traffic by an open space or barrier)
- [ ] Combination of bicycle lane and unsigned shared roadway
- [ ] Combination of unsigned shared roadway and signed shared roadway
- [ ] None
- [ ] Other (please specify)

* 56. Please rank these route attributes from the most important (1) to the least important (7) to you.

- Good pavement
- Avoid big uphills
- Travel time
- Avoiding stop signs and/or stoplights
- Adequate lighting
- Safe from motor vehicles on the roadway
- Safety from crime

* 57. My usual route is relatively flat with few hills and valleys.

- Strongly agree
- Agree
- Neutral
- Disagree
- Strongly disagree

* 58. There is not much motor vehicle traffic on my usual route.

- Strongly agree
- Agree
- Neutral
- Disagree
- Strongly disagree

* 59. I am comfortable cycling in mixed traffic.

- Strongly agree
- Agree
- Neutral
- Disagree
- Strongly disagree

Impact of Air Pollution Information on Bicycle Route Choice: Commuter

* 60. There are lots of trees, gardens, parks, or interesting architecture and buildings along my usual route.

- Strongly agree
- Agree
- Neutral
- Disagree
- Strongly disagree

* 61. The noise level on my usual route is high.

- Strongly agree
- Agree
- Neutral
- Disagree
- Strongly disagree

* 62. I believe the presence of bus and truck traffic makes a route unsafe for bicyclists.

- Strongly agree
- Agree
- Neutral
- Disagree
- Strongly disagree

Impact of Air Pollution Information on Bicycle Route Choice: Commuter

63. Please leave any additional comments that you think would be helpful for the researchers.

If you would like to know about the findings of the study, please contact us at sabreena.anowar@utoronto.ca.
