# Supplementary material for: Accommodating exogenous variable and decision rule heterogeneity in discrete choice models: Application to bicyclist route choice
Source: PLoS One. 2018 Nov 30;13(11):e0208309. doi: 10.1371/journal.pone.0208309 (PMC6268012; doi:10.1371/journal.pone.0208309)
Supplement: S1 File — (PDF) [file pone.0208309.s011.pdf]

UNIVERSITY OF TORONTO

OFFICE OF THE VICE-PRESIDENT, RESEARCH AND INNOVATION

PROTOCOL REFERENCE # 32793

April 6, 2016

Dr. Marianne Hatzopoulou
DEPT OF CIVIL ENGINEERING
FAC OF APPLIED SCI & ENG

Dear Dr. Hatzopoulou,

Re: Your research protocol entitled, "Evaluating the acute cardiovascular health effects of particulate air pollution using personal and mobility-based exposure assessment"

ETHICS APPROVAL

Original Approval Date: April 6, 2016
Expiry Date: April 5, 2017
Continuing Review Level: 1

We are writing to advise you that the Health Sciences Research Ethics Board (REB) has granted approval to the above-named research protocol under the REB's delegated review process. Your protocol has been approved for a period of **one year** and ongoing research under this protocol must be renewed prior to the expiry date.

Any changes to the approved protocol or consent materials must be reviewed and approved through the amendment process prior to its implementation. Any adverse or unanticipated events in the research should be reported to the Office of Research Ethics as soon as possible.

Please ensure that you submit an Annual Renewal Form or a Study Completion Report 15 to 30 days prior to the expiry date of your current ethics approval. Note that annual renewals for studies cannot be accepted more than 30 days prior to the date of expiry.

If your research is funded by a third party, please contact the assigned Research Funding Officer in Research Services to ensure that your funds are released.

Best wishes for the successful completion of your research.

Yours sincerely,

Research Oversight and Compliance Office - Human Research Ethics Program
McMurrich Building, 12 Queen's Park Crescent West, 2nd Floor, Toronto, ON M5S 1S8 Canada
Tel: +1 416 946-3273 ● Fax: +1 416 946-5763 ● ethics.review@utoronto.ca ● http://www.research.utoronto.ca/for-researchers-administrators/ethics/

Elizabeth Peter, Ph.D.
REB Chair

UNIVERSITY OF TORONTO

OFFICE OF THE VICE-PRESIDENT, RESEARCH AND INNOVATION

PROTOCOL REFERENCE # 32793

March 13, 2017

Dr. Marianne Hatzopoulou
DEPT OF CIVIL ENGINEERING
FAC OF APPLIED SCI & ENG

Dear Dr. Hatzopoulou,

Re: Your research protocol entitled, "Evaluating the acute cardiovascular health effects of particulate air pollution using personal and mobility-based exposure assessment"

**ETHICS APPROVAL**	**Original Approval Date: April 6, 2016**<br>**Expiry Date: April 5, 2018**<br>**Continuing Review Level: 1**<br>**Renewal: Data Analysis Only**

We are writing to advise you that you have been granted annual renewal of ethics approval to the above-referenced research protocol through the Research Ethics Board (REB) delegated process. Please note that all protocols involving ongoing data collection or interaction with human participants are subject to re-evaluation after 5 years. Ongoing research under this protocol must be renewed prior to the expiry date.

Any changes to the approved protocol or consent materials must be reviewed and approved through the amendment process prior to its implementation. Any adverse or unanticipated events should be reported to the Research Oversight and Compliance - Human Research Ethics Program as soon as possible. If your research is funded by a third party, please contact the assigned Research Funding Officer in Research Services to ensure that your funds are released.

Please ensure that you submit an Ethics Renewal Form or a Study Completion/Closure Report 15 to 30 days prior to the expiry date of your protocol. Note that ethics renewals for studies cannot be accepted more than 30 days prior to the date of expiry as per our guidelines.

Please note, all approved research studies are eligible for a routine Post-Approval Review (PAR) site visit. If chosen, you will receive a notification letter from our office. For information on PAR, please see http://www.research.utoronto.ca/wp-content/uploads/documents/2014/09/PAR-Program-Description-1.pdf.

Best wishes for the successful completion of your research.

Yours sincerely,

Elizabeth Peter

Elizabeth Peter, Ph.D.
REB Chair
